# The *De Novo* Assembly of Mitochondrial Genomes of the Extinct Passenger Pigeon (*Ectopistes migratorius*) with Next Generation Sequencing

**DOI:** 10.1371/journal.pone.0056301

**Published:** 2013-02-20

**Authors:** Chih-Ming Hung, Rong-Chien Lin, Jui-Hua Chu, Chia-Fen Yeh, Chiou-Ju Yao, Shou-Hsien Li

**Affiliations:** 1 Bell Museum and Department of Ecology, Evolution, and Behavior, University of Minnesota, Saint Paul, Minnesota, United States of America; 2 Department of Life Science, National Taiwan Normal University, Taipei, Taiwan; 3 Department of Biology, National Museum of Natural Science, Taichung, Taiwan; University of York, United Kingdom

## Abstract

The information from ancient DNA (aDNA) provides an unparalleled opportunity to infer phylogenetic relationships and population history of extinct species and to investigate genetic evolution directly. However, the degraded and fragmented nature of aDNA has posed technical challenges for studies based on conventional PCR amplification. In this study, we present an approach based on next generation sequencing to efficiently sequence the complete mitochondrial genome (mitogenome) of two extinct passenger pigeons (*Ectopistes migratorius*) using *de novo* assembly of massive short (90 bp), paired-end or single-end reads. Although varying levels of human contamination and low levels of postmortem nucleotide lesion were observed, they did not impact sequencing accuracy. Our results demonstrated that the *de novo* assembly of shotgun sequence reads could be a potent approach to sequence mitogenomes, and offered an efficient way to infer evolutionary history of extinct species.

## Introduction

Species extinction is a process that prunes the tree of life [1] and eliminates the genetic information harbored by the species. Ancient DNA (aDNA) extracted from fossils or museum specimens can provide information to help us position extinct species in the tree of life and investigate evolution of genes directly [2], [3]. For example, aDNA was used to clarify the phylogenetic relationships among enigmatic extinct dodos (*Raphus cucullatus*) and other Columbidae species [4], to assess whether there were functional differences in mitochondrial DNA leading to different fates between two clades of woolly mammoths (*Mammuthus primigenius*) [5], and to estimate the evolutionary rate of base substitutions in the mitochondrial DNA of Adélie penguins (*Pygoscelis adeliae*) [6]. Studies based on aDNA could also be important for conservation by providing insights into the historical demographics of extinct populations or species and possible causes of their extinction [7]. For example, results of aDNA analyses suggested an association between the extinction of Beringian steppe bison (*Bison priscus*) and the onset of the last glacial cycle [8].

Polymerase chain reaction (PCR) amplification has been widely used to amplify targeted regions from aDNA [4], [9], [10]. However, owing to the highly degraded and fragmented nature of aDNA, researchers need to amplify and sequence numerous overlapping short fragments to assemble sequences of sufficient lengths for analysis. The small quantity and poor quality of aDNA also result in high rates of PCR failure [11]. These difficulties make mitochondrial genomes (mitogenomes) a main target for PCR-based aDNA analyses because of their high copy numbers relative to loci in the nuclear genome and the availability of more published references for primer design than for nuclear loci [12], [13]. However, nuclear copies of mitochondrial genes (numts) [14] or contamination by exogenous DNAs can produce non-target PCR products [13]. It is especially severe when conserved PCR primers preferentially amplify numts instead of authentic mitochondrial fragments [15]. Another potential challenge in aDNA analyses is that the postmortem instability of nucleotides can cause miscoding lesions during PCR amplifications [16]. The predominant type of postmortem damage in aDNA is cytosine (C) to thymine (T) transitions, which lead to guanine (G) to adenine (A) transitions on the complementary strand, due to the deamination of cytosines [17]. These biases can result in misleading inferences of demographic histories in aDNA studies using conventional PCR amplification [18].

More recently, high-throughput sequencing technologies (i.e., the next generation sequencing or NGS), capable of yielding up to millions of short sequences simultaneously, are ideal for sequencing fragmented DNA and help overcome challenges encountered in aDNA studies [3], [19]. Due to the high copy numbers of mitochondria in eukaryotic cells, a few gigabases (Gbp or 10^9^ base pairs) of NGS data should be sufficient to recover an entire mitogenome with a great sequencing depth. High sequencing depths of NGS can reduce the impact of numts or other non-target products in yielding incorrect sequences [13], [20]. Furthermore, high-throughput haploid mitogenomes are ideal for estimating postmortem damage of aDNA by excluding the confounding effect of heterozygosity in diploid genomes [11]. In this study, we recovered the entire mitogenome of an extinct avian species, the passenger pigeon (*Ectopistes migratorius*), from aDNA extracted from two museum skin specimens using *de novo* assembly of NGS reads.

Passenger pigeons were one of the most abundant avian species ever recorded, but they went extinct over a brief period of time between the late 19^th^ and early 20^th^ century [21]. Although the extinction of the passenger pigeon has been mainly attributed to anthropogenic causes [22], its historical population dynamics have been debated [23]. Analyses based on aDNA provide an opportunity to infer the historical population dynamics of the passenger pigeon. In this study, we sequenced the mitogenomes of two passenger pigeons by directly shotgun sequencing aDNA without a PCR-based target enrichment protocol, which was often adopted in aDNA studies targeting on mitogenomes [13], [19], [24], [25]. The elimination of PCR enrichment saves time and also avoids the practical problems associated with aDNA amplification, such as a lack of long templates for PCR and increased chances of contamination by modern DNA [19].

## Materials and Methods

### Historical DNA Extraction and Quantification

Toe pads (approximately 5×2×2 mm) were excised from two passenger pigeon specimens archived in the Bell Museum of Natural History at the University of Minnesota (one male, BMNH1149, and one female, BMNH1389, collected at Grand Marais, Cook Co., Minnesota in July and August 1879, respectively). The toe pad tissue was cut into small pieces, washed with 100% ethanol twice, and placed in 100% ethanol overnight. The wash procedure was repeated with 70% ethanol the next day and finally the tissue was washed twice with ddH_2_O. These washing steps could remove microbes and artificial materials from the surfaces of museum samples and gradually hydrate the samples so that they can be more efficiently digested by proteinase *K* [26]. The Qiagen DNeasy Tissue Kit (Qiagen) was used to extract DNA with the following modifications: (1) 180 µl ATL buffer and 20 µl proteinase *K* solution (600 mAU/ml) was added to the samples, which were rotated in an incubator in 56°C for 24 h. (2) An additional 20 µl proteinase *K* solution was added to the sample rotated in 56°C for another 24 h. (3) 100 µl preheated (70°C) ddH_2_O, instead of AE buffer, was used to elute DNA from the spin column twice, resulting in a total volume of 200 µl. We performed the DNA extraction inside a fume hood in a room used for plant genetic research to prevent contamination from other avian species. DNA extract was quantified by PicoGreen assay at the BioMedical Genomics Center of the University of Minnesota. Concentrations of both aDNA samples were approximately 2 ng per µl.

### Illumina Genomic Sequencing, de novo Assembly, and Annotation

Approximately 250 and 400 ng of aDNA, extracted from passenger pigeons BMNH1149 and BMNH1138, respectively, were used for paired-end shotgun sequencing (Paired-end DNA sample preparation kit, Illumina) following instructions by the manufacturer. We did not perform a prior PCR-based target enrichment protocol. The prior DNA fragmentation for library preparation was also omitted because of the fragmented nature of aDNA. DNA fragments of 200±50 bp (base pair) were selected to ligate with paired-end adaptors for the subsequent PCR amplification. Paired-end 90 bp sequence reads (i.e., 2×90 bp in the pair) were obtained from a separate lane of a HiSeq 2000 Genome Analyzer (Illumina) for each sample. The paired-end library preparation and sequencing were performed by BGI (Beijing Genomic Institute, Shenzhen, China).

We used the “Remove Duplicate Read” tool implemented in the CLC Genomics Workbench version 5.0 (CLC Bio) to remove duplicate reads. To reconstruct mitogenome sequence of the passenger pigeon, we took advantage of the high copy numbers of mitogenomes to assemble non-duplicate paired-end reads *de novo* using the “De Novo Assembly” tool in the CLC Genomics Workbench. We set the costs of mismatch, insertion and deletion to 2, 3 and 3, respectively. Parameters of similarity and overlap were set to 0.97 and 0.5, respectively. Only contigs longer than or equal to 300 bp were used for subsequent analyses. We also *de novo* assembled the paired-end reads as single-end reads to test whether shorter read length reduced assembling efficiency.

*De novo* assembled contigs greater than 10 Kbp were blasted against the NCBI nr database using the “BLAST” tool implemented in the CLC Genomics Workbench. The cutoff E-value of 1.0E-15 was used and only the best 33 hits for each query were collected. To annotate the *de novo* assemblies, we aligned the putative mitogenomes of passenger pigeons with the published complete mitochondrial genome of the domestic pigeon (or rock pigeon), *Columba livia* (GenBank accession number: GU908131) [27] with the aid of “Alignment” tool implemented in the CLC Genomic Workbench with the default setting.

### Assay of Exogenous Contamination

From sample collecting, specimen preparing, to archiving, the odds that museum skin specimens are contaminated by exogenous sources, such as human, fungal or bacterial DNA, can be high. To assay how many of the shotgun sequence reads are of an avian origin and how many are exogenous DNA, we downloaded chicken (*Gallus gallus*), human (*Homo sapiens*), (73 complete) fungal and (3853 complete) bacterial genomes from the NCBI genome database and used the “Map Reads to Reference” tool in the CLC Genomics Workbench to map non-duplicate reads to these reference genomes. Because the whole genome data of the passenger pigeon or other Columbiformes species were not available, we used the proportion of the reads matching the chicken genome as an indicator of the proportion of our samples belonging to the passenger pigeon. The costs of mismatch, insertion and deletion were set the same as those used in the *de novo* assembling, and the parameters of similarity and overlap for mapping were set to 0.9 (except 0.97 for the human genome so that we could specifically identify the level of contamination with human DNA) and 0.5, respectively.

### Assay of Postmortem Damage in Passenger Pigeon aDNA

Sequence error rates were estimated by comparing raw reads with the consensus sequences of the assembled mitogenomes [11], [28]. The error rate was defined as the number (bp) of mismatches between raw reads and consensus sequences divided by the total amount (bp) of raw reads. Because the mitochondrial genome is haploid, the observed discrepancies between raw reads and consensus sequences are attributed to postmortem damage, PCR errors during library construction, or sequencing errors. If postmortem damage is the main factor contributing to the errors, the transitions from C to T and from G to A should be the predominant types of mismatches [16].

### Comparison of Mitogenomes among Passenger Pigeons

We aligned the sequences of the three available mitogenomes of the passenger pigeon, which included two genomes from this study and one unpublished genome from the NCBI GenBank (accession number JQ692598 by Novak BJ; the latter was extracted from FMNH4797 [voucher number of the Field Museum] collected at Troy, Rensselaer Co., New York in 1860) using the CLC Genomic Workbench to preliminarily assess the mitogenomic variation of this extinct species.

## Results and Discussion

### *De novo* Assemblies of Passenger Pigeon Mitogenomes

The sequencing qualities of the 90 bp reads were generally high for both passenger pigeon samples. 97.5% of the reads in BMNH1149 and 93.6% of the reads in BMNH1389 passed Q20, which indicates the probability of a base call error ≤0.01 (Table 1). After removing duplicate reads, we used approximately 3.5 and 4.7 Gbp of sequences from BMNH1149 and BMNH1389, respectively, for subsequent analyses.

**Table 1:** Sequence information for paired-end reads by illumina genomic sequencing of two passenger pigeons.

Voucher	Read	% Q20	Duplicate	Assembled Contig
BMNH1149	42,135,314	97.5	2,824,624	368,118
BMNH1389	64,100,016	93.6	12,109,764	161,686

Read indicates the total numbers of 90 bp reads, % Q20 indicates the percentages of reads passing Q20 (the probability of a base call error ≤0.01), duplicate indicates the number of duplicate reads removed and assembled contig indicates the number of assembled contigs ≥300 bp.

**Figure 1:**
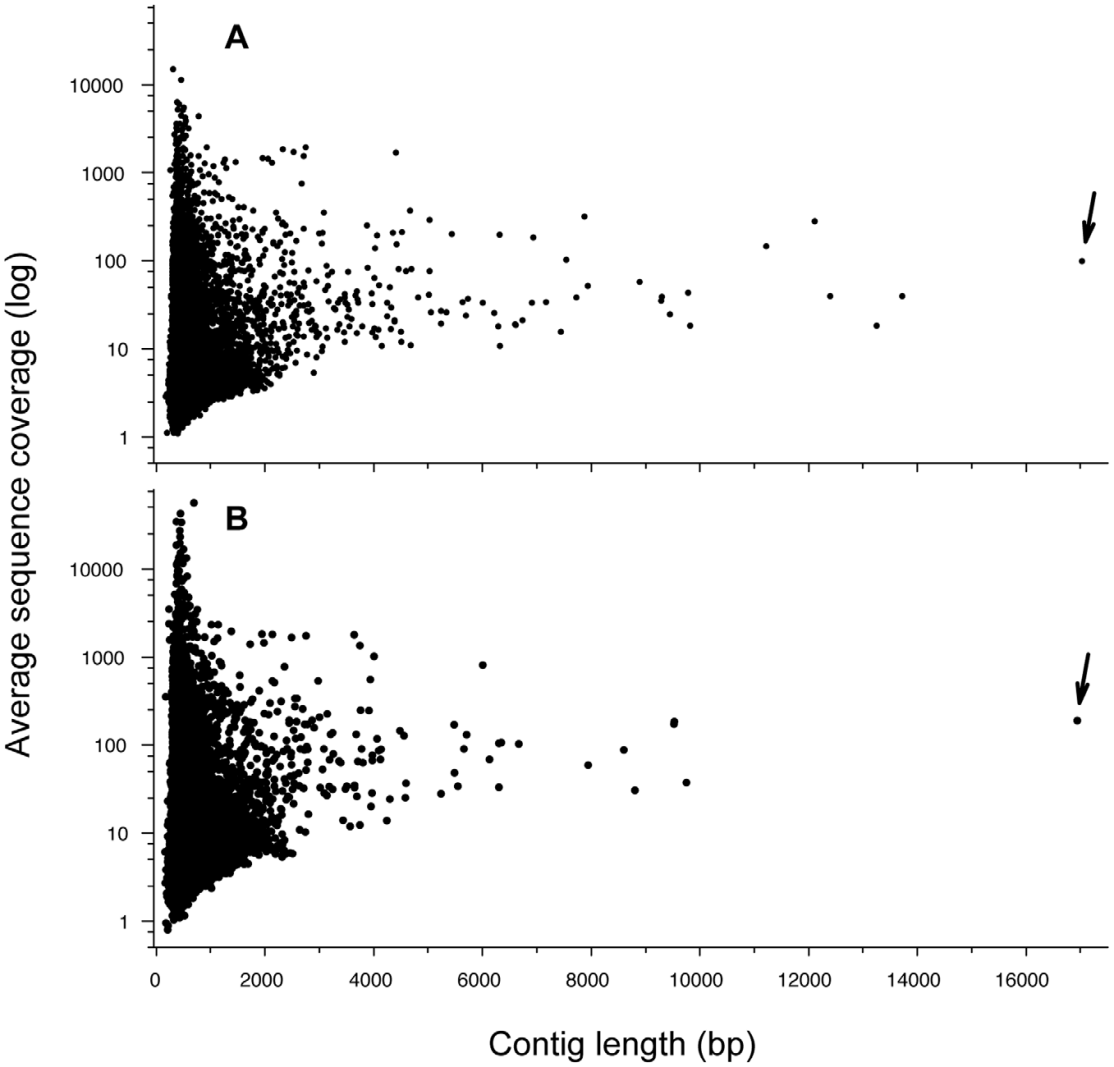
Average sequence coverage of contigs against their lengths (bp) for the *de novo* assemblies based on Illumina paired-end reads of (A) BMNH1149 and (B) BMNH1389. Arrows indicate the contigs that were annotated as the mitogenomes of passenger pigeons.

Assuming the haploid genome size of the passenger pigeon is similar to those of other Columbiformes birds (1.1–1.6 Gbp; Animal Genome Size Database) [29], the expected sequence depths for the genomes of both passenger pigeons sequenced are around 2.2–4.3. However, if the levels of exogenous contamination are high in the aDNA samples, the expected sequence depths can be significantly reduced. There were in total 368,118 and 161,686 contigs assembled *de novo* based on the paired-end reads for BMNH1149 and BMNH1389, respectively (Table 1). We found that sequencing depths (the average sequence coverage) of the assembled contigs were generally small: the mode of average sequence coverage was 4.0 for the contigs of both samples. 88.9% and 61.1% of the contigs assembled for BMNH1149 and BMNH1389, respectively, had sequencing depths less than five (Figure 1). Only 0.4% of the contigs in BMNH1149 and 1.5% in BMNH1389 had sequencing depths equal to or greater than 50. The lengths of most contigs (98.7% and 98.5% in BMNH1149 and BMNH1389, respectively) were less than 1 Kbp (Figure 1). Only six BMNH1149 contigs and one BMNH1389 contig had lengths longer than 10 Kbp.

**Figure 2:**
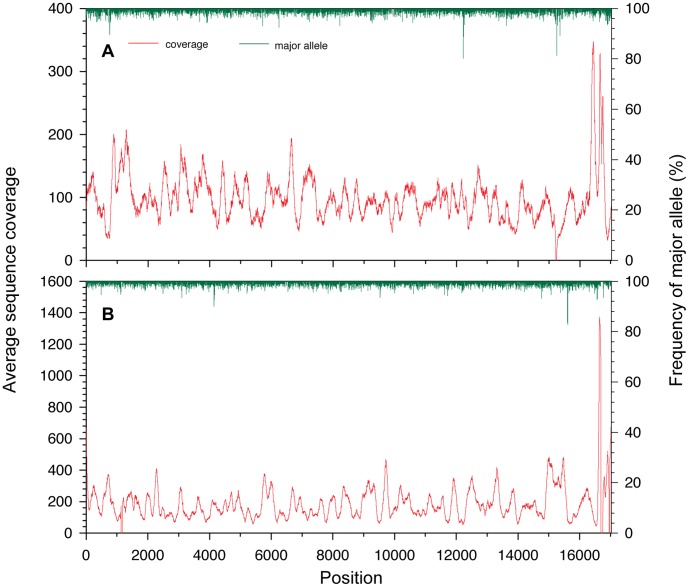
Sequence coverage (in red) and frequency of the major allele (in green) of each base pair along the mitogenomes of BMNH1149 (upper) and BMNH1389 (lower). Both mitogenomes start from the first base of tRNA-Phe.

The blast results indicated that the top five hits (E-value = 0) of the longest contig in each sample (17,026 and 16,943 bp for BMNH1149 and BMNH1389, respectively) were the mitogenomes of five Columbiformes species (data not shown) including the unpublished passenger pigeon mitogenome (16,715 bp). Therefore, the longest contig in each sample was highly likely to be the complete or nearly complete mitogenome of the passenger pigeon (NBCI accession numbers KC489473-489474). We found that the average sequence depths of these two contigs (97.7 and 187.6 for BMNH1149 and BMNH1389, respectively) were about 25 to 47 times higher than the mode of the average sequence depths of all contigs (Figure 1). The high sequencing depths of the longest contigs are consistent with the high copy numbers of mitochondria in eukaryotic cells and indirectly confirm the sequences’ mitochondrial origin. In addition, the average sequence depths along the mitogenomes varied considerably (Figure 2). We also found that the other five long contigs (>10 Kbp) in BMNH1149 were homologous to the chicken genome (data not shown).

The *de novo* assemblies based on single-end reads (90 bp) produced similar results to those based on paired-end reads (90×2 bp). For example, the longest contigs based on single-end reads were 15,547 and 15,526 bp, which were also best blasted to the mitogenomes of five Columbiformes species, for BMNH1149 and BMNH1389, respectively. The results suggested that the *de novo* assembles based on single-end reads can still produce quality mitogenome sequences. Thus this approach is an efficient way to sequence mitogenomes from poor-quality DNA sources.

### Characterization of Passenger Pigeon Mitogenomes

The passenger pigeon mitogenomes assembled in this study were similar to those of the domestic pigeon [27] and other Columbiformes species (e.g., *Chalcophaps indica* and *Leptotila verreauxi*) [30] containing 13 protein-coding genes, 22 tRNAs, two rRNAs and the control region (see the alignment of passenger pigeon and domestic pigeon mitogenomes in Figure S1). There were no internal stop codons found in the protein-coding genes. The gene order of the passenger pigeon mitogenome was identical to those of Columbiformes species [27], [30]. The nucleotide composition of two mitogenome assemblies was not highly biased (G : A : T : C = 0.14∶ 0.30∶ 0.25∶ 0.31 for both assemblies). Two to four alleles were detected at 5,023 and 7,276 sites of the BMNH1149 and BMNH1389 mitogenome assemblies, respectively. Nevertheless, the frequencies of the major alleles at these sites were high (mean = 0.985±0.01 [S.D.], range: 0.802 to 0.997 for BMNH1149; mean = 0.989±0.008 [S.D.], range: 0.828 to 0.999 for BMNH1389; Figure 2). The dramatically low frequencies of minor alleles indicate that these sites are homozygous and these minor alleles can be caused by postmortem damages in DNA, sequencing or assembling errors. The lack of heterozygous sites suggests that these assemblies have an origin of mitochondria because mitochondrial sequences do not have heterozygous positions unless heteroplasmy occurs.

The ambiguous sites (a total of 20 and 42 Ns in BMNH1149 and BMNH1389, respectively; Figure S1), shown in the alignment of the two *de novo* mitogenome assemblies, are probably sequencing errors associated with homopolymers or repetitive DNA sequences. For instance, a stretch of 20 Ns was flanked by 12 and 18 uninterrupted Cs within the 16 s rRNA of the BMNH1149 mitogenome (Figure S1). However, the homologous region in the BMNH1389 mitogenome included only 12 uninterrupted Cs. Therefore, the stretch of Ns and additional 18 Cs could be artifacts caused by homopolymer sequencing or assembling errors. In addition, two stretches of ambiguous sequences (20 and 22 Ns) in the control region of the BMNH1389 mitogenome were embedded within homologous regions composed by AAC/GTT repeats in the BMNH1419 mitogenome (Figure S1).

### Varying Levels of Exogenous Contamination in Passenger Pigeon aDNA

**Figure 3:**
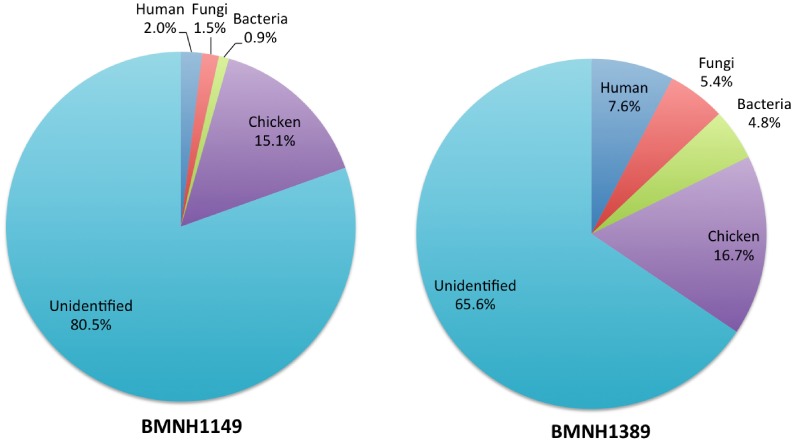
Metagenomic composition of the Illumina sequence reads from the toe pads of two passenger pigeons, BMNH1149 and BMNH1389. Percentage of non-duplicate reads blasted to chicken, human bacterial and fungal genome references downloaded from the NCBI genome database.

Our results indicated that the levels of contamination varied between the two aDNA samples: 2.0% and 7.6% of all non-duplicate reads could be mapped to the human reference genome, 1.5% and 5.4% to the fungal genomes, and 0.9% and 4.8% to the bacterial genomes for BMNH1149 and BMNH1389, respectively (Figure 3). Other studies reported varying levels of contamination with human DNA (0.14%–9.8%)[11], [31]–[34] and microbial DNA (0.01%–45%) [11], [28], [31], [34] in aDNA samples, and the contamination levels in this study were within the reported ranges. Thus, our results are consistent with the findings in other studies that exogenous contamination are common for aDNA. However, the contamination should have little impact on accuracy of the consensus sequence in this study.

There were 15.1% and 16.7% of reads blasted to the chicken genome and 80.5% and 65.6% of reads unidentified for BMNH1149 and BMNH1389, respectively (Figure 3). Although the unidentified rates in our samples were higher than those reported in other aDNA studies (5.5%–19.4%) [11], [28], [31], we believe that the higher rates were due to the lack of reference genomes from closely related species in this study. Thus, the results imply that the proportion of reads belonging to the passenger pigeon should be higher than 15%, shown by the blasting results to the chicken genome, in both samples. Furthermore, the higher proportion of exogenous sequences, higher redundancy of raw reads and fewer *de novo* assembled contigs indicate that the quality of aDNA from the toe pads of BMNH1389 was poorer than that of BMNH1149, although we do not currently understand the reason(s). Nevertheless, variable qualities among samples are common in aDNA studies [20], [32].

### Low Postmortem Damage in Passenger Pigeon aDNA

**Figure 4:**
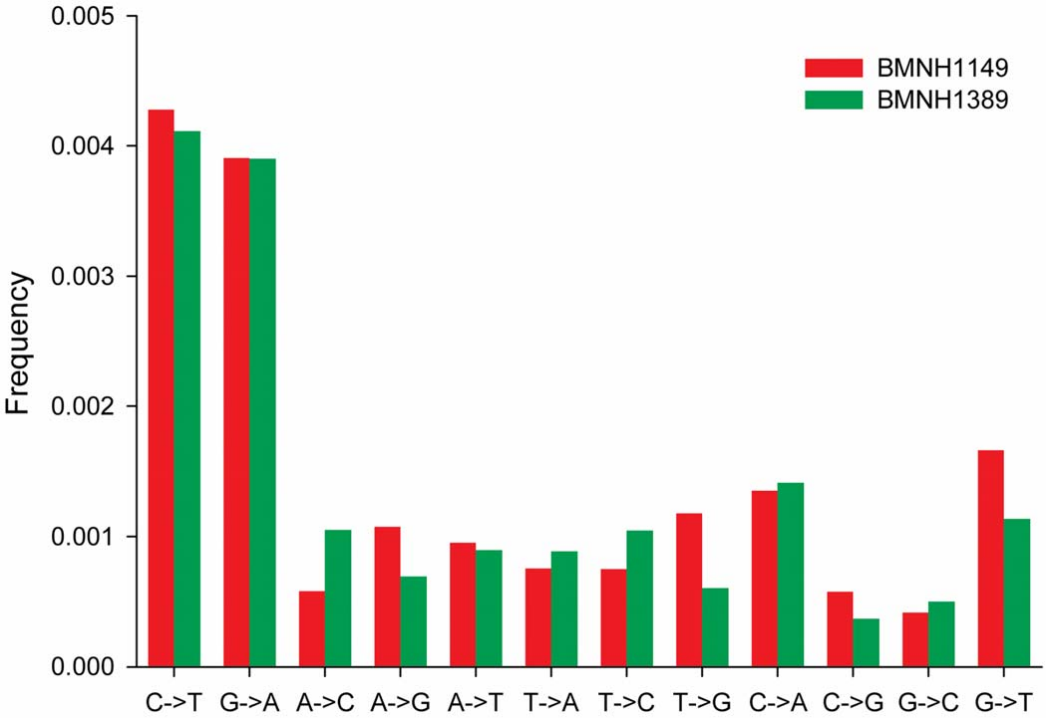
Frequency for 12 categories of sequence mismatches for raw read sequences against the consensus sequence of mitogenomes of BMNH1149 (in green) and BMNH1389 (in red).

The levels of nucleotide mismatch between the raw read sequences and the consensus sequences were generally low (Figure 4). A total of 6,995 and 13,032 mismatches were found out of 1,663,480 and 3,178,345 bases of raw reads in BMNH1149 and BMNH1389, respectively. The error rates were about 0.004, similar to the reported sequencing error of Illumina technology [35], [36]. We divided these mismatches into 12 types (all possible directions of mismatches among four different nucleotides), and found that frequencies of C to T and G to A transitions were 2.5 to 11.1 times higher than those of other types of mismatches for the two mitogenome assemblies (Figure 4). We did not observe similar mismatch rates for T to C and A to G transitions (Figure 4), suggesting that postmortem damage might play a role in elevating the level of putative transitions [16]. Nevertheless, the frequency of these two types of mismatch combined was less than 1% (Figure 4), indicating that postmortem damage would have had limited effect on the chemical integrity of our aDNA samples.

### Comparison of Mitogenomes among Passenger Pigeons

The 16S rRNA and control region of the unpublished passenger pigeon mitogenome deposited in the GenBank (JQ692598) had more gaps when aligned to the domestic pigeon mitogenome (176 [bp of gaps]/1633 [bp of alignment length] and 422/1679 for the 16S rRNA and control region, respectively) than those of the two *de novo* assembled mitogenomes reported in this study (36/1633 and 58/1633, and 245/1779 and 329/1679 for the 16S rRNA and control region, respectively; Table S1 and Figure S1). In addition, the ND6 gene in JQ692598 had one stretch of 12 ACC-repeats and one of a long poly-A (Figure S1), which might be caused by sequencing or assembling errors (as we discussed above). A similar pattern, in which more mismatched sites were associated with JQ692598 than the other two passenger pigeon samples in the 16S rRNA, ND6 and control region, was also observed in the pairwise comparisons among the three passenger pigeon mitogenomes (Table 2). It may signal poor sequencing quality of these regions in JQ692598 as well as in our samples. There were 14 segregating sites among the three aligned passenger pigeon mitogenomes excluding the 16S rRNA, ND6 and control region where numerous gaps and potentially erroneous sequences were found (Table 2). Among them, four are nonsynonymous substitutions. However, our sample size was too small to infer their historical population size and any temporal changes. For the future work, we will sequence mitogenomic and nuclear data from a larger number of passenger pigeons to infer their historical population dynamics.

**Table 2:** Pairwise divergence among the three passenger pigeon mitogenomes.

	tRNA-Phe		12S rRNA			tRNA-Val	16S rRNA		tRNA-Leu1		ND1			tRNA-Ile				tRNA-Gln
Alignment length	69		972			73	1633		74		966			71				71
BMNH1149/BMNH1389	0/NA/0/0		0/NA/0/0			0/NA/0/0	3/NA/27/20		0/NA/0/0		1/1/0/0			0/NA/0/0				0/NA/0/0
BMNH1149/JQ692598	2/NA/1/0		0/NA/0/0			0/NA/0/0	**32/**NA**/148/**20		0/NA/0/0		1/1/0/0			0/NA/0/0				0/NA/0/0
BMNH1389/JQ692598	2/NA/1/0		0/NA/0/0			0/NA/0/0	**33/**NA**/168/**0		0/NA/0/0		0/0/0/0			0/NA/0/0				0/NA/0/0
	**tRNA-Met**		**ND2**			**tRNA-Trp**	**tRNA-Ala**		**tRNA-Asn**		**tRNA-Cys**			**tRNA-Tyr**				**COX1**
Alignment length	69		1042			71	69		69		67			72				1551
BMNH1149/BMNH1389	0/NA/0/0		1/0/1/0			0/NA/0/0	0/NA/0/0		0/NA/0/0		0/NA/0/0			0/NA/0/0				2/1/0/0
BMNH1149/JQ692598	0/NA/0/0		2/1/1/0			0/NA/0/0	0/NA/0/0		0/NA/0/0		0/NA/0/0			0/NA/0/0				0/0/0/0
BMNH1389/JQ692598	0/NA/0/0		1/1/1/0			0/NA/0/0	0/NA/0/0		0/NA/0/0		0/NA/0/0			0/NA/0/0				2/1/0/0
	**tRNA-Ser1**		**tRNA-Asp**	**COX2**			**tRNA-Lys**		**ATP8**			**ATP6**		**COX3**				**tRNA-Gly**
Alignment length	74		69	684			71		168			684		784				69
BMNH1149/BMNH1389	0/NA/0/0		0/NA/0/0	0/0/0/0			0/NA/0/0		0/0/0/0			0/0/0/0		1/0/0/0				0/NA/0/0
BMNH1149/JQ692598	0/NA/0/0		0/NA/0/0	0/0/0/0			0/NA/0/0		0/0/0/0			0/0/0/0		0/0/0/0				0/NA/0/0
BMNH1389/JQ692598	0/NA/0/0		0/NA/0/0	0/0/0/0			0/NA/0/0		0/0/0/0			0/0/0/0		1/0/0/0				0/NA/0/0
	**ND3**	**tRNA-Arg**			**ND4L**			**ND4**		**tRNA-His**			**tRNA-Ser2**		**tRNA-Leu2**		**ND5**	
Alignment length	352	69			297			1378		69			66		71		1815	
BMNH1149/BMNH1389	0/0/0/0	0/NA/0/0			0/0/0/0			0/0/0/0		0/NA/0/0			0/NA/0/0		0/NA/0/0		5/1/0/0	
BMNH1149/JQ692598	0/0/0/0	0/NA/0/0			0/0/0/0			0/0/0/0		0/NA/0/0			0/NA/0/0		0/NA/0/0		5/1/0/0	
BMNH1389/JQ692598	0/0/0/0	0/NA/0/0			0/0/0/0			0/0/0/0		0/NA/0/0			0/NA/0/0		0/NA/0/0		0/0/0/0	
	**CYTB**	**tRNA-Thr**			**tRNA-Pro**			**ND6**		**tRNA-Glu**			**Control region**					
Alignment length	1143	68			70			522		71			1679					
BMNH1149/BMNH1389	2/0/0/0	0/NA/0/0			0/NA/0/0			1/0/0/0		0/NA/0/0			4/NA/310/0					
BMNH1149/JQ692598	2/0/0/0	0/NA/0/0			0/NA/0/0			**23/12/**0/0		0/NA/0/0			**143/**NA**/406/**3					
BMNH1389/JQ692598	2/0/0/0	0/NA/0/0			0/NA/0/0			**24/12**/0/0		0/NA/0/0			**1421/**NA**/489/**3					

We divided the mitogenome into regions encoding 22 transfer RNA genes (Phenylalanine [tRNA-Phe], Valine [tRNA-Val], Leucine [tRNA-Leu], Isoleucine [tRNA-Ile], Glutamine [tRNA-Gln], Methionine [tRNA-Met], Tryptophan [tRNA-Trp], Alanine [tRNA-Ala], Asparagine [tRNA-Asn], Cysteine [tRNA-Cys], Tyrosine [tRNA-Tyr], Serine [tRNA-Ser], Asparate [tRNA-Asp], Lysine [tRNA-Lys], Glycine [tRNA-Gly], Arginine [tRNA-Arg], Histidine [tRNA-His], Serine [tRNA-Ser], Leucine [tRNA-Leu], Threonine [tRNA-Thr], Proline [tRNA-Pro], Glutamate [tRNA-Glu]), 13 protein-coding genes (*NADH dehydrogenase subunit 1* [ND1], *NADH dehydrogenase subunit 2* [ND2], *cytochrome c oxidase subunit I* [COX1], *cytochrome c oxidase subunit II* [COX2], *ATP synthase F0 subunit 8* [ATP8], *ATP synthase F0 subunit 6* [ATP6], *cytochrome c oxidase subunit III* [COX3], *NADH dehydrogenase subunit 3* [ND3], *NADH dehydrogenase subunit 4L* [ND4L], *NADH dehydrogenase subunit 4* [ND4], *NADH dehydrogenase subunit 5* [ND5], *cytochrome b* [CYTB], *NADH dehydrogenase subunit 6* [ND6]), two ribosomal RNA genes (12 s rRNA and16 s rRNA) and a control region. Alignment length (bp), and number of segregating sites/number of nonsynonymous substitutions/number of gaps/number of ambiguous sites are shown for each of these regions. Numbers that are exceptionally high were bolded.

### Applications of the de novo Assembly of Shotgun Sequence Reads

Our results demonstrated that the *de novo* assembly of short reads generated by shotgun sequencing could be an efficient approach to sequence mitogenomes from aDNA. Our approaches have advantages over others including (1) conventional PCR, (2) multiplex PCR [37], (3) conventional or multiplex PCR amplification followed by NGS [38] and (4) probe capture followed by NGS [39]. Those approaches require prior genetic information, which is often unavailable in extinct species, for primer design, whereas no prior information is required to our approach for both sequencing and assembling procedures. It is also difficult to design primers for short DNA templates [19]. The approach based on conventional PCR requires a fair amount of template DNA that is often limited in ancient samples. Around 10–50 ng of template DNA are needed for each conventional PCR and hundreds of reactions are needed to assemble a mitogenome of 17 Kbp assuming a mean length of 200 bp or shorter for aDNA fragments. By contrast, our approach only needs 300 ng of DNA to sequence a mitogenome assuming that the level of exogenous DNA in a sample is not excessive. Even though multiplex PCR requires less template DNA, designing primers for simultaneously amplifying numerous fragments in a single PCR reaction is never trivial [13]. The susceptibleness of aDNA to PCR contamination by modern DNA is also a concern. Although probe capture combined with NGS can sequence fairly short fragments and reduce the risk of PCR contamination, the initial cost for setting up a probe capture system is high [13] and the loss for template DNA during library preparation is considerable [19]. The omission of DNA fragmentation during library preparation in our approach prevents substantial loss of template DNA. However, one disadvantage of our approach is that the current cost of sequencing a large amount of mitogenomes is prohibitive for many laboratories (i.e., 1–2 thousand dollars per sample). A temporary strategy is to apply our approach to sequence one mitogenome of study species and base on the sequence information to perform multiplex PCR amplification or probe capture followed by NGS to pooled samples each of which is uniquely barcoded using tagged primers [40]. Of course, this strategy works only if the technological obstacles of multiplex PCR can be overcome or the initial costs of setting up a probe system is affordable. It is clear that as the cost of NGS is decreasing rapidly, our approach will soon be the simplest and even most affordable method for sequencing mitogenomes. Our approach can also assess the levels of exogenous contamination in samples and potentially produce sequence data of parasites and pathogens in focal species for relevant studies.

Although we only employed this approach to toe pads in this study, it can potentially be applied to other ancient tissues such as other soft tissues, bones, hairs, feathers, or eggshells. Soft tissues have high levels of endogenous DNA, but their aDNA tend to be more fragmented than other tissues’ [11], [13]. This study demonstrates that mitogenomes can be assembled based on single-end reads as short as 90 bp. Thus, our approach should work to many ancient soft tissues. Bones tend to retain long sequence fragments yet contain abundant exogenous DNA [13]. We applied this approach to a whale bone stored in a museum but a high level of exogenous DNA prevented us from *de novo* assembling its mitogenome (data not shown). Thus, target enrichment before sequencing or higher sequencing depths may be needed for ancient bones that contain abundant exogenous DNA. Hairs, feathers and eggshells are found to have long fragments and less exogenous DNA [20], [41], [42], and thus our approach can be suitable to these samples.

## Supporting Information

Figure S1 **Mitogenome alignment of one domestic pigeon (Kan et al. 2010. GenBank accession no. GQ240309) and three passenger pigeons (one deposited in the GenBank, JQ692598, by Novak and two reported in the current study with museum IDs, BMNH1149 and BMNH1389).** A base identical to that of the domestic pigeon is shown as a dot. A gap is indicated by a dash. An IUCN ambiguity code is colored in red. Annotation of the domestic pigeon mitogenome is positioned along sequences and labeled within flags. The relative position of the last base of each line on the corresponding mitogenome is indicated at the end of each line.
(PDF)

Table S1 **Mitogenomic divergence between the rock pigeon (** ***Columba livia*** **, GenBank accession number GU908131) and each of the three passenger pigeons (one unpublished sequence with a GenBank accession number JQ692598 and two sequenced in this study).** We divided the mitogenome into regions encoding 22 transfer RNA genes (Phenylalanine [tRNA-Phe], Valine [tRNA-Val], Leucine [tRNA-Leu], Isoleucine [tRNA-Ile], Glutamine [tRNA-Gln], Methionine [tRNA-Met], Tryptophan [tRNA-Trp], Alanine [tRNA-Ala], Asparagine [tRNA-Asn], Cysteine [tRNA-Cys], Tyrosine [tRNA-Tyr], Serine [tRNA-Ser], Asparate [tRNA-Asp], Lysine [tRNA-Lys], Glycine [tRNA-Gly], Arginine [tRNA-Arg], Histidine [tRNA-His], Serine [tRNA-Ser], Leucine [tRNA-Leu], Threonine [tRNA-Thr], Proline [tRNA-Pro], Glutamate [tRNA-Glu]), 13 protein-coding genes (*NADH dehydrogenase subunit 1* [ND1], *NADH dehydrogenase subunit 2* [ND2], *cytochrome c oxidase subunit I* [COX1], *cytochrome c oxidase subunit II* [COX2], *ATP synthase F0 subunit 8* [ATP8], *ATP synthase F0 subunit 6* [ATP6], *cytochrome c oxidase subunit III* [COX3], *NADH dehydrogenase subunit 3* [ND3], *NADH dehydrogenase subunit 4L* [ND4L], *NADH dehydrogenase subunit 4* [ND4], *NADH dehydrogenase subunit 5* [ND5], *cytochrome b* [CYTB], *NADH dehydrogenase subunit 6* [ND6]), two ribosomal RNA genes (12 s rRNA and16 s rRNA), a control region and the remaining intergenic noncoding regions. For each of these regions, its alignment length (bp) and number of segregating sites/number of gaps/number of ambiguous sites are shown. Numbers that are extraordinarily high or low were bolded.
(DOCX)
