# Supplementary material for: The De Novo Assembly of Mitochondrial Genomes of the Extinct Passenger Pigeon (Ectopistes migratorius) with Next Generation Sequencing
Source: PLoS One. 2013 Feb 20;8(2):e56301. doi: 10.1371/journal.pone.0056301 (PMC3577829; doi:10.1371/journal.pone.0056301)
Supplement: Figure S1 — Mitogenome alignment of one domestic pigeon (Kan et al. 2010. GenBank accession no. GQ240309) and three passenger pigeons (one deposited in the GenBank, JQ692598, by Novak and two reported in the current study with museum IDs, BMNH1149 and BMNH1389). A base identical to that of the domestic pigeon is shown as a dot. A gap is indicated by a dash. An IUCN ambiguity code is colored in red. Annotation of the domestic pigeon mitogenome is positioned along sequences and labeled within flags. The relative position of the last base of each line on the corresponding mitogenome is indicated at the end of each line. (PDF) [file pone.0056301.s001.pdf]

```
                    tRNA-Phe                                                          12S rRNA
Pigeon (GU908131)  GTCCCCGTAGCTTACAATCAAAGCATGGCACTGAAGACGCCAAGACGGCCCTACACTCACCC-GGGGACAAAAGACTTAG  79
JQ692598           A-...T...........-...................T...........T.G...TC.....CA................  78
BMNH1149           .....T...........-...................T...........T.G...TC.....CA................  79
BMNH1389           .....T...........-...................T...........T.G...TC.....CA................  79

                   12S rRNA
Pigeon (GU908131)  TCCTAACCTTACCATTAGTTCTTGCTAGATATATACATGCAAGTATCCGCACTCCAGTGTAAATGCCCTTGACTCCTACG  159
JQ692598           ....................T....C...C.................T...TC.................A........A  158
BMNH1149           ....................T....C...C.................T...TC.................A........A  159
BMNH1389           ....................T....C...C.................T...TC.................A........A  159

                   12S rRNA
Pigeon (GU908131)  CCGTAGGTAAAAGGAGCCGGCATCAGGCACGCCCATG-GCAGCCCAAGACGCCTTGCTTAGCCACACCCCCACGGGTACT  238
JQ692598           TT...............T..T...........T...AA....................C.....................  238
BMNH1149           TT...............T..T...........T...AA....................C.....................  239
BMNH1389           TT...............T..T...........T...AA....................C.....................  239

                   12S rRNA
Pigeon (GU908131)  CAGCAGTAATTAACATTAAGCAATGAGTGTAAACTTGACTTAGTCATAGCAACTCAGGGTTGGTAAATCTTGTGCCAGCC  318
JQ692598           ........................A.......G..............G................................  318
BMNH1149           ........................A.......G..............G................................  319
BMNH1389           ........................A.......G..............G................................  319

                   12S rRNA
Pigeon (GU908131)  ACCGCGGTCACACAAGAGACCCAAGCTAATCGTTTGCGGCGTAAAGAGTGGACTCATGCTTATCACACTAATTAAGGTCA  398
JQ692598           ........................AT........C..................C.....C.....T.TC.....G...T.  398
BMNH1149           ........................AT........C..................C.....C.....T.TC.....G...T.  399
BMNH1389           ........................AT........C..................C.....C.....T.TC.....G...T.  399

                   12S rRNA
Pigeon (GU908131)  AAACGCAACTGAGCTGTCATAAGCCTAAGATGCATTTAAAACCGCCCTAAAGATGACCCTAAATTCACAAGACTTAATGA  478
JQ692598           .......G................T....G...GC........A.........C........-.CT.T.T....A.C.A.  477
BMNH1149           .......G................T....G...GC........A.........C........-.CT.T.T....A.C.A.  478
BMNH1389           .......G................T....G...GC........A.........C........-.CT.T.T....A.C.A.  478

                   12S rRNA
Pigeon (GU908131)  ACTCCACGAAAGCCAGGGCACAAACTGGGATTAGATACCCCACTATGCCTGGCCCTAAATCTTGATGCTCTATATAACCA  558
JQ692598           ..................T..................................................-..C.C.....  556
BMNH1149           ..................T..................................................-..C.C.....  557
BMNH1389           ..................T..................................................-..C.C.....  557

                   12S rRNA
Pigeon (GU908131)  AAGCATCCGCCTGAGAACTACGAGCACAAACGCTTAAAACTCTAAGGACTTGGCGGTGCCCCAAACCCACCTAGAGGAGC  638
JQ692598           ................................................................................  636
BMNH1149           ................................................................................  637
BMNH1389           ................................................................................  637
```

```
                   12S rRNA
Pigeon (GU908131)  CTGTTCTGTAATCGATACTCCACGATACACCCGACCACTTCTTGCCATGGACAGCCTACATACCGCCGTCGCCAGCTCAC 718

JQ692598           ....C..A.........AC.......................A....-AA.............................. 715

BMNH1149           ....C..A.........AC.......................A....-AA.............................. 716

BMNH1389           ....C..A.........AC.......................A....-AA.............................. 716
                   12S rRNA
Pigeon (GU908131)  CTCCTCTGAGAGCACTACAGTGAGCACAACCGCCCTAACCCCGCTAACAAGACAGGTCAAGGTATAGCTTATGAAGTGGA 798

JQ692598           ...TC........G................T...............G.....................CC.......... 795

BMNH1149           ...TC........G................T...............G.....................CC.......... 796

BMNH1389           ...TC........G................T...............G.....................CC.......... 796
                   12S rRNA
Pigeon (GU908131)  AGAAATGGGCTACATTTTCTAATCTAGAAAATCCC-ACGAAAAGGGGCATGAAATAGCCCCTAGAAGGCGGATTTAGCAG 877

JQ692598           ...G..................C......T...T.T...G..G...........C......C.................. 875

BMNH1149           ...G..................C......T...T.T...G..G...........C......C.................. 876

BMNH1389           ...G..................C......T...T.T...G..G...........C......C.................. 876
                   12S rRNA
Pigeon (GU908131)  TAAAGAGGGATAATATAAGCCCCCTTTAAGCTGGCTCTGGGGCACGTACATACCGCCCGTCACCCTCCTCATAAGCTCTA 957

JQ692598           .....C...........T.............C.......A..T............................C........ 955

BMNH1149           .....C...........T.............C.......A..T............................C........ 956

BMNH1389           .....C...........T.............C.......A..T............................C........ 956
                   12S rRNA
Pigeon (GU908131)  AACTACCAATAAATAATACTCCTACCTGCTAAAGATGAGGTAAGTCGTAACAAGGTAAGTGTACCGGAAGGTGCACTTAG 1037

JQ692598           G..........G.......AA...TT...................................................... 1035

BMNH1149           G..........G.......AA...TT...................................................... 1036

BMNH1389           G..........G.......AA...TT...................................................... 1036
                   12S rRN tRNA-Val                                                      16S rRNA
Pigeon (GU908131)  CACACCAAGACGTAGCTACAATGT-AAAGCATTCAGCTTACACCTGAAAGATGCCTGCCACACACCAGGTCGTCTTGAAG 1116

JQ692598           T.................T..CA.G................................T....T................. 1115

BMNH1149           T.................T..CA.G................................T....T................. 1116

BMNH1389           T.................T..CA.G................................T....T................. 1116
                   16S rRNA
Pigeon (GU908131)  CCAACTCTAGCCCAACCCCCC-------------------------------------ACGACAACCGCACATAAAAATC 1159

JQ692598           .............CC......---------------------------------------A....--A....A.C...-- 1152

BMNH1149           .............CC......CNNNNNNNNNNNNNNNNNNNNCCCCCCCCCCCCCCCCC.AT...--A..TG........ 1194

BMNH1389           .............CC......C--------------------------------------AT...--A..TG........ 1156
                   16S rRNA
Pigeon (GU908131)  TACTAAATCTTTAAATCAAAACATTCTTCCAACTAAGTATAGGCGATAGAAAAGTCATTAGGCGCTATAGAGATCTGTAC 1239

JQ692598           -.AA..CCACCC...----------------...T................T.....C.......G........TC.... 1215

BMNH1149           .....G.CACC....CT.........C..TG...T................T.....C.......G........TC.... 1274

BMNH1389           .....G.CACC....CT.........C..TG...T................T.....C.......G........TC.... 1236
```

```
                  16S rRNA
Pigeon (GU908131) CGCAAGGGAGAGATGAAATAACAATGAACTAAGCCGAGCAATAAACAGCAAAGATAAACCCTTGTACCTCTTGCATTATG 1319
         JQ692598 ..T......A..........G..G....-......A....GC..............G...................C... 1294
         BMNH1149 ..T......A..........G..G....-......A....GC..............G...................C... 1353
         BMNH1389 ..T......A..........G..G....-......A....GC..............G...................C... 1315
                  16S rRNA
Pigeon (GU908131) GTCTAGCTAGAATAACCAAGCAAAGTGGACTTAAGCTTGCCACCCCGAAACCTAAGCGAGCTACTTGTAAGCAGCTAACC 1399
         JQ692598 A...........C.........G..C...................................................C.. 1374
         BMNH1149 A...........C............C...................................................C.. 1433
         BMNH1389 A...........C............C...................................................C.. 1395
                  16S rRNA
Pigeon (GU908131) CTGAGCGAACCCGTCTCTGTTGCAAAAGAGTGGGACGACTTACTAGTAGAGGTGAAAAGCCAATCGAGCTAGGTGATAGC 1479
         JQ692598 ...................................T..................................G......... 1454
         BMNH1149 ...................................T..................................G......... 1513
         BMNH1389 ...................................T..................................G......... 1475
                  16S rRNA
Pigeon (GU908131) TGGTTACCTGCAAAACGAATCTAAGTTCACCCTTGACACCTCCTCCAGGAGCCCTTACCACAACCCCAATGTAGGTCATC 1559
         JQ692598 ...........G...T.............A........T...........A..T.C.--G.C.......C.A..A.AG.. 1532
         BMNH1149 ...........G...T.............A........T...........A..T.C.--G.C.......C.A..A.AG.. 1591
         BMNH1389 ...........G...T.............A........T...........A..T.C.--G.C.......C.A..A.AG.. 1553
                  16S rRNA
Pigeon (GU908131) AAGAGTAATTTAAAGGAGGTACAGCTCCTTTAAAAAAGGATACAACCTTCACTAGCGGATAATTACAGCCTTA--CATAA 1637
         JQ692598 .....C..........G........C......................C.T...........C..ATAA..C.TG..C.. 1612
         BMNH1149 .....C..........G........C......................C.T...........C..ATAA..C.TG..C.. 1671
         BMNH1389 .....C..........G........C......................C.T...........C..ATAA..C.TG..C.. 1633
                  16S rRNA
Pigeon (GU908131) CAATTGTGGGCCTTTAAGCAGCCATCAGTAAAGAGTGCGTCAAAGCTCCCCTTT-ACAAAAATCCTAAATCCGTATGACT 1716
         JQ692598 ........................C..AC...................TA...CC............G.C.ACC.C.... 1692
         BMNH1149 ........................C..AC...................TA...CC............G.C.ACC.C.... 1751
         BMNH1389 ........................C..AC...................TA...CC............G.C.ACC.C.... 1713
                  16S rRNA
Pigeon (GU908131) CCCTCCCCACTAGCAGGTCAACCTATACTAATAGGAGAGTTAATGCTAAAATGAGTAACTAGGGCAAACCCCCTCTTAAG 1796
         JQ692598 ....T.T.....T.....T..T......CC....A...A.........................-G..........A... 1771
         BMNH1149 ....T.T.....T.....T..T......CC....A...A.........................-G..........A... 1830
         BMNH1389 ....T.T.....T.....T..T......CC....A...A.........................-G..........A... 1792
                  16S rRNA
Pigeon (GU908131) CGCAAACTTACATCTACACATTATTAACAGGCACCTAATACTCACACTACAACAAGACTACGTATTACATCCCCTGTTAT 1876
         JQ692598 ..............CT..............A..........A.TA.TCT.........C.T.....TT.C.........A 1851
         BMNH1149 ..............CT..............A..........A.TA.TCT.........C.T.....TT.C.........A 1910
         BMNH1389 ..............CT..............A..........A.TA.TCT.........C.T.....TT.C.........A 1872
```

	16S rRNA	
Pigeon (GU908131)	CCCAACCCAGGAGCGCATGCTAGAAAGATTGAAATCTGTAAAAGGAACTAGGCAAATCTCCAAGGCCCGACTGTTTACCA	1956
JQ692598	................C.A...........T..........................A..-...................	1930
BMNH1149	................C.A...........T..........................A..-...................	1989
BMNH1389	................C.A...........T..........................A..-...................	1951
	16S rRNA	
Pigeon (GU908131)	AAAACATAGCCTTCAGCCAAAC-GAGTATTGAAGGTGATGCCTGCCCAGTGACTT--TGTTCAACGGCCGCGGTATCCTA	2033
JQ692598	...................G..CA...............................CG.......................	2010
BMNH1149	...................G..CA...............................CG.......................	2069
BMNH1389	...................G..CA...............................CG.......................	2031
	16S rRNA	
Pigeon (GU908131)	ACCGTGCGAAGGTAGCGCAACCAATTGTCTCATAAATTGAGACTTGTATGAATGGCTAAACGAGGTCTTAACTGTCTCTT	2113
JQ692598	....................T........C.......C..	2090
BMNH1149	....................T........C.......C..	2149
BMNH1389	....................T........C.......C..	2111
	16S rRNA	
Pigeon (GU908131)	GCAGATAATCAGTGAAATTGATCTTCCTGTGCAAAAGCAGGAATAAACGCATAAGACGAGAAGACCCTGTGGAACTTAAA	2193
JQ692598	A...G.G.A...............................	2170
BMNH1149	A...G.G.A...............................	2229
BMNH1389	A...G...A...............................	2191
	16S rRNA	
Pigeon (GU908131)	AATCAGCAGCCACCTCGCAACAAAACTTAACCTACTAGGCTCACCA--GACATGATGCTGGCTCGCATTTTTCGGTTGGG	2271
JQ692598	................AT........CC.-..C..CG...C....GCCA..CAACAA.----------------------	2227
BMNH1149	................AT........CC.-..C..CG...C.....CCA...AAGC........................	2308
BMNH1389	................AT........CC.-..C..CG...C.....CCA...AAGC.......T................	2270
	16S rRNA	
Pigeon (GU908131)	GCGACCTTGGAGAAAAACAAAACCTCCAAAAATAAGACCACCCCT-CTTAACTAAGAGCAACCCCTCGACG-ACTAACAG	2349
JQ692598	------------............AAA.....A..A..A.---------...A.CA.-----------------------	2263
BMNH1149	.....................T..........C........TT..T......C......T.......A...T.....T..	2388
BMNH1389	.....................T...................TT..T......C......T.......A...T.....T..	2350
	16S rRNA	
Pigeon (GU908131)	TAACCAGGACCCAATATAATTGACCAATGGACCAAGCTACCCCAGGGATAACAGCGCAATCTCCTCCAAGAGTCCATATC	2429
JQ692598	-.....A-.....CA.----------------------------------...AAA................C.......	2307
BMNH1149	.......-..C.......	2467
BMNH1389	.......-..C.......	2429
	16S rRNA	
Pigeon (GU908131)	GACGAGGAGGTTTACGACCTCGATGTTGGATCAGGACATCCTAATGGTGCAGCCGCTATCAAGGGTTCGTTTGTTCAACG	2509
JQ692598	....T....................	2387
BMNH1149	....T....................	2547
BMNH1389	....T....................	2509

```
                      16S rRNA
Pigeon (GU908131)   ATTAATAGTCCTACGTGATCTGAGTTCAGACCGGAGCAATCCAGGTCGGTTTCTATCTATGTCGTACTTTTCCCAGTACG 2589
JQ692598            ..............................................................T.A........T...... 2467
BMNH1149            ..............................................................T.A........T...... 2627
BMNH1389            ..............................................................T.A........T...... 2589
                      16S rRNA
Pigeon (GU908131)   AAAGGACCGGAAAAGTGAGGCCAATGATTCAACACATGCCTCCTCCCTAAGTAATGAATACAACTAAATTACAAAGAGAT 2669
JQ692598            ................AG........C......GT.C...C....T...........CC..................... 2547
BMNH1149            ................AG........C......GT.C...C....T...........CC..................... 2707
BMNH1389            ................AG........C......GT.C...C....T...........CC..................... 2669
                      16S rRNA                        tRNA-Leu
Pigeon (GU908131)   CCCACACAGTACCCAATCCTAGAAAAGGATCGCTAGCGTGGCAGAGTTTGGTAAATGCAAAAGGCTTAAGCCCTTTACCC 2749
JQ692598            ........AC...T..................................C............................T.. 2627
BMNH1149            ........AC...T..................................C............................T.. 2787
BMNH1389            ........AC...T..................................C............................T.. 2749
                    tRNA-Leu                                 ND1
Pigeon (GU908131)   AGAGGTTCAAGTCCTCTCCCTAGCTCACTACCTTCCATGTTTATAACCTACCTCATTATATCACTATCGTACGCAGTACC 2829
JQ692598            ..........A.................C...--.....A.C....T......T.....G..C.....A........... 2705
BMNH1149            ..........A.................C...--.....A.C...GT......T.....G..C.....A........... 2865
BMNH1389            ..........A.................C...--.....A.C....T......T.....G..C.....A........... 2827
                    ND1
Pigeon (GU908131)   AATCCTAATCGCCGTAGCATTCCTGACTCTAGTGGAACGAAAAGTTCTAAGCTACATGCAAGCTCGAAAAGGCCCTAACA 2909
JQ692598            .........T........G...T.A..A.....A..G..C......T..........A...T.......G.....A.... 2785
BMNH1149            .........T........G...T.A..A.....A..G..C......T..........A...T.......G.....A.... 2945
BMNH1389            .........T........G...T.A..A.....A..G..C......T..........A...T.......G.....A.... 2907
                    ND1
Pigeon (GU908131)   TTGTAGGGCCTTTCGGGCTCCTCCAACCAGTGGCAGATGGTGTCAAACTATTCATTAAAGAACCCATCCGCCCATCCACC 2989
JQ692598            ..........C.....A..A..T........A...........A..G................................A 2865
BMNH1149            ..........C.....A..A..T........A...........A..G................................A 3025
BMNH1389            ..........C.....A..A..T........A...........A..G................................A 2987
                    ND1
Pigeon (GU908131)   TCCTCCCCAATTCTTTTTATCATGACCCCCATTCTAGCCCTGCTCTTAGCCATTACAATCTGAATTCCTCTCCCCCTCCC 3069
JQ692598            .....T.................A.....T..A.....T..T..T.......................C........T.. 2945
BMNH1149            .....T.................A.....T..A.....T..T..T.......................C........T.. 3105
BMNH1389            .....T.................A.....T..A.....T..T..T.......................C........T.. 3067
                    ND1
Pigeon (GU908131)   CTTCTCCCTCACCGACCTTAACTTGGGCCTCCTCTTTCTTCTAGCCATATCAAGCCTAGCAGTCTACTCCATCCTATGAT 3149
JQ692598            ......T..T..A.........C.A.....T.....C..C........................A..T..........G. 3025
BMNH1149            ......T..T..A.........C.A.....T.....C..C........................A..T..........G. 3185
BMNH1389            ......T..T..A.........C.A.....T.....C..C........................A..T..........G. 3147
```

```
                   ND1
Pigeon (GU908131)  CTGGCTGAGCCTCCAACTCAAAGTACGCACTCATTGGGGCACTACGAGCAGTAGCACAAACCATCTCCTATGAAGTAACA 3229
JQ692598           ..........T..T........A.....T........T........G.................T.....C........C 3105
BMNH1149           ..........T..T........A.....T........T........G.................T.....C........C 3265
BMNH1389           ..........T..T........A.....T........T........G.................T.....C........C 3227

                   ND1
Pigeon (GU908131)  CTAGCCATCATTCTTCTATCCGTGATTATATTAAGTGGAAATTACACTCTCAATACCCTTGCCACCACCCAAGAACCCAT 3309
JQ692598           ..............C........A...........C.....C..T..C..T..C........T.TT...........A.. 3185
BMNH1149           ..............C........A...........C.....C..T..C..T..C........T.TT...........A.. 3345
BMNH1389           ..............C........A...........C.....C..T..C..T..C........T.TT...........A.. 3307

                   ND1
Pigeon (GU908131)  ATACCTCATCTTCTCCTCCTGACCCCTCGCAATAATGTGATACATCTCCACCCTTGCCGAAACTAATCGAGCCCCATTCG 3389
JQ692598           ...T..A...........T.....A..A.....G..A........T.................C..C...........T. 3265
BMNH1149           ...T..A...........T.....A..A.....G..A........T.................C..C...........T. 3425
BMNH1389           ...T..A...........T.....A..A.....G..A........T.................C..C...........T. 3387

                   ND1
Pigeon (GU908131)  ACCTTACCGAAGGAGAATCCGAACTAGTCTCTGGCTTCAACGTAGAATATGCCGCAGGCCCATTCGCCCTTTTCTTCCTG 3469
JQ692598           .T..C..A...........T...........A.....T...........C..T.....T....................A 3345
BMNH1149           .T..C..A...........T...........A.....T...........C..T.....T....................A 3505
BMNH1389           .T..C..A...........T...........A.....T...........C..T.....T....................A 3467

                   ND1
Pigeon (GU908131)  GCCGAATATGCTAACATCATACTAATAAACACCATAACTGCCATCCTCCTCCTAAACCCAAGTTCATTAAACCTACCCCC 3549
JQ692598           ...........A..T..T...T...................T..T..AT.............C...............T. 3425
BMNH1149           ...........A..T..T...T...................T..T..AT.............C...............T. 3585
BMNH1389           ...........A..T..T...T...................T..T..AT.............C...............T. 3547

                   ND1
Pigeon (GU908131)  TGAACTATACCCCCTAATTCTAGCCACAAAAACTCTTCTTCTCTCCTCTGGATTCCTTTGAGTCCGTGCCTCTTACCCAC 3629
JQ692598           A..G....T....GC.G.A....................C..A..T..A...........GA....C..T.......... 3505
BMNH1149           A..G....T....GC.G.A....................C..A..T..A...........GA....C..T.......... 3665
BMNH1389           A..G....T....GC.G.A....................C..A..T..A...........GA....C..T.......... 3627

                   ND1
Pigeon (GU908131)  GATTCCGCTACGATCAGCTCATACATCTCCTCTGAAAAAATTTCCTACCCTTAACCCTAGCCCTATGCCTATGACACACT 3709
JQ692598           .............C..A..T.....C.....A........C...T....AC....A..............T........C 3585
BMNH1149           .............C..A..T.....C.....A........C...T....AC....A..............T........C 3745
BMNH1389           .............C..A..T.....C.....A........C...T....AC....A..............T........C 3707

                   ND1                                                                tRNA-Ile
Pigeon (GU908131)  AGCATGCCCATCTCCTATGCAGGCCTTCCACCCTACCTAAGAAAAACACCCCAACAAATGGAAATGTGCCTGAACTCAAG 3789
JQ692598           .....A........A..C...............C..............A------G..C...............T.T... 3659
BMNH1149           .....A........A..C...............C..............A------G..C...............T.T... 3819
BMNH1389           .....A........A..C...............C..............A------G..C...............T.T... 3781
```

```
                    tRNA-Ile                                                                        tRNA-Gln
Pigeon (GU908131)   GGTCACTATGATAAAGTGAACATAGAGGTACACTAACCCTCTCATTTCCTTCACCTTAGAAAAGCAGGATTCGAACCTAC 3869
         JQ692598   ................................................................T............... 3739
         BMNH1149   ................................................................T............... 3899
         BMNH1389   ................................................................T............... 3861

                                                                tRNA-Met
                                                           tRNA-Gln
Pigeon (GU908131)   ACAAAAGAGATCAAAACTCCTCATACTTCCTCTATATTATTTTCTAGCAGGGTCAGCTAATAAAGCTATCGGGCCCATAC 3949
         JQ692598   ...................TCT......................................CG.................. 3819
         BMNH1149   ...................TCT......................................CG.................. 3979
         BMNH1389   ...................TCT......................................CG.................. 3941

                    tRNA-Met
                                                   ND2
Pigeon (GU908131)   CCCGAAAATGATGGTTTAACCCCTTCCCCTACTAATGAACCCACACGCCATATTAGTTTCAACCCTAAGCCTACTCTTAG 4029
         JQ692598   ................................................A..G..TA.C......T.C.........C... 3899
         BMNH1149   ................................................A..G..TA.C......T.C.........C... 4059
         BMNH1389   ................................................A..G..TA.C......T.C.........C... 4021

                    ND2
Pigeon (GU908131)   GAACAACCATCACCATTTCAAGCAACCACTGGGTCATAGCTTGAACCGGACTAGAAATTAACACTCTTGCAATCATCCCA 4109
         JQ692598   .......T.......................A..............T...........C.....C..............C 3979
         BMNH1149   .......T.......................A..............T...........C.....C..............C 4139
         BMNH1389   .......T.......................A..............T...........C.....C..............C 4101

                    ND2
Pigeon (GU908131)   TTTATCTCCGAACCTCACCACCCACGAGCTATTGAAGCCACAGTCAAATACTTCCTAGTACAAGCAACAGCATCACCCCT 4189
         JQ692598   ..C.....AA....C..............C...........GA....G..T........................G.... 4059
         BMNH1149   ..C.....AA....C..............C...........GA....G..T........................G.... 4219
         BMNH1389   ..C.....AA....C..............C...........GA....G..T........................G.... 4181

                    ND2
Pigeon (GU908131)   ACTCTTATTCTCAAGTATGTCCAATGCCTGAGCCACTGGACAATGAGATATTACCCAACTCACCCACCCAACATCATGCA 4269
         JQ692598   ......G........C..A........T....................C..............................C 4139
         BMNH1149   ......G........C..A........T....................C..............................C 4299
         BMNH1389   ......G........C..A........T....................C..............................C 4261

                    ND2
Pigeon (GU908131)   TTCTACTTACAATTGCAATCTCCATAAAGCTAGGACTAGTACCATTCCACTTTTGATTCCCAGAAGTACTTCAAGGTTCA 4349
         JQ692598   .C..G........C...G..........A.........................................A.....C... 4219
         BMNH1149   .C..G........C...G..........A.........................................A.....C... 4379
         BMNH1389   .C..G........C...G..........A.........................................A.....C... 4341
```

	ND2	
Pigeon (GU908131)	TCCATAACCACAGCACTACTACTATCCACAGCCCTAAAACTTCCCCCAATTACCATCCTCCTCATAACATCCCACTCACT	4429
JQ692598	C......TA..T.....G........A..T.T...T...T.C......G.CG.T..............C.....T.....	4299
BMNH1149	C......TA..T.....G........A..T.T...T...T.C......G.CG.T..............C.....T.....	4459
BMNH1389	C......TA..T.....G........A..T.T...T...T.C......G.CG.T..............C.....T.....	4421
	ND2	
Pigeon (GU908131)	AAACCCAACTCTACTGACCATTATGGCTATCTCCTCAGCAGCCCTGGGAGGCTGAATAGGACTCAATCAAACTCAAATCC	4509
JQ692598	........T.A....A....CC..A..C...G.T.........T.A..G...........C..A..C..G..A..G.CT.	4379
BMNH1149	........T......A....CC..A..C...G.T.........T.A..G...........C..A..C..G..A..G.CT.	4539
BMNH1389	........T......A....CC..A..C...G.T.........T.A..G...........C..A..C..G..A..G.CT.	4501
	ND2	
Pigeon (GU908131)	GAAAAATCTTAGCCTTCTCCTCCATCTCCCACATAGGATGAATAGTGGTCATCATCATTTACAACCCAAACCTCACCCTT	4589
JQ692598	........C.............A........T.....T.......CCA.......T..C..T.........T.A.....A	4459
BMNH1149	........C.............A........T.....T.......CCA.......T..C..T.........T.A.....A	4619
BMNH1389	........C.............A........T.....T.......CCA.......T..C..T.........T.A.....A	4581
	ND2	
Pigeon (GU908131)	CTAACCTTCTACCTCTATACCCTTATAACCACCACTGTATTCCTCACTCTTAGCACCACTAAAACACTAAAACTAACAAC	4669
JQ692598	.....T........A..C.T...A.....TT....C..........TC..C.A...A..C...G...........T....	4539
BMNH1149	.....T........A..C.T...A.....TT....C..........TC..C.A...A..C...G...........T....	4699
BMNH1389	.....T........A..C.T...A.....TT....C..........TC..C.A...A..C...G...........T....	4661
	ND2	
Pigeon (GU908131)	AATGATAACCTCGTGAACAAAAACCCCCATACTAACCGCAACATTAATAATAACCCTACTCTCACTAGCGGGCCTTCCAC	4749
JQ692598	...A....T...A..............A...T...A......CC...........T....T........A..........	4619
BMNH1149	...A....T...A..............A...T...A......CC...........T....T........A..........	4779
BMNH1389	...A....T...A..............A...T...A......CC...........T....T........A..........	4741
	ND2	
Pigeon (GU908131)	CACTAACAGGCTTCTTACCTAAATGACTCATCATTCAAGAGCTTACCAAGCGAGAAATAACCTTAACAGCCACAATCATG	4829
JQ692598	.............TC....A..G...........C........C..T..A.AG............G....T.........	4699
BMNH1149	.............TC....A..G...........C........C..T..A.AG............G....T........A	4859
BMNH1389	.............TC....A..G...........C........C..T..A.AG............G....T.........	4821
	ND2	
Pigeon (GU908131)	GCTATGCTTTCTCTACTTGGGCTATTCTTCTACCTCCGCCTTGCATACTACTCGACAATCACTCTGCCCCCCAACACTAC	4909
JQ692598	..CT.A..A..C.....C..A..............T........G..T.....A........C..A..A........C..	4779
BMNH1149	..CT.A..A..C.....C..A..............T........G..T.....A........C..A..A........C..	4939
BMNH1389	..CT.A..A..C.....C..A..............T........G..T.....A........C..A..A........C..	4901
	ND2	
Pigeon (GU908131)	AAACCACATAAAACAGTGGCACACCAACAAAACCACAAGCACCCCAGTTGCCATCCTAACCTCATTAGCCACCCTGCTCC	4989
JQ692598	....T..........A..A..T..T.....G..A....A.....T.A.....C.......T..TC..A..G....A....	4859
BMNH1149	....T..........A..A..T..T.....G..A....A.....T.A.....C.......T..TC..A..G....A....	5019
BMNH1389	....T..........A..A..T..T.....G..A....A.....T.A.....C.......T..TC..A..G....A....	4981

```
                     ND2                                  tRNA-Trp
Pigeon (GU908131)  TGCCACTCTCCCCCATAATTCTCACAACCCTTTAAGAAACTTAGGATCGACCCAAACCAAAGGCCTTCAAAGCCTTAGAC 5069
JQ692598           .T...........T.....C...........C..-.................T........................A.. 4938
BMNH1149           .T...........T.....C...........C..-.................T........................A.. 5098
BMNH1389           .T...........T.....C...........C..-.................T........................A.. 5060

                   tRNA-Trp                                                            tRNA-Ala
Pigeon (GU908131)  AAGAGTTAAACCCTCTTGGTTTCTGCTAAGACTCGCAGGACATTAACCTGCATCCCCCGAATGCAACTCAGATGCTTTAA 5149
JQ692598           .................A.............T..........C............TTT............A......... 5018
BMNH1149           .................A.............T..........C............TTT............A......... 5178
BMNH1389           .................A.............T..........C............TTT............A......... 5140

                   tRNA-Ala                                                            tRNA-Asn
Pigeon (GU908131)  TTAAGCTAGAACCTTGACTAGACAGATGGGCCTCGATCCCATAAACTCCTGGTTAACAGCCAGACGCCTAAACCAACAGG 5229
JQ692598           ...............AG..............T............G..................G.............G.. 5098
BMNH1149           ...............AG..............T............G..................G.............G.. 5258
BMNH1389           ...............AG..............T............G..................G.............G.. 5220

                   tRNA-Asn                                                            tRNA-Cys
                                                                                       tRNA-Tyr
Pigeon (GU908131)  CTTCTATCTATTAGACTCCGGCACTCTTAACGTGCATCAATGAGCTTGCGACTCAACATGAAACTTTCACTACAGAGTCG 5309
JQ692598           ..........CC.....................................A.............................. 5178
BMNH1149           ..........CC.....................................A.............................. 5338
BMNH1389           ..........CC.....................................A.............................. 5300

                                                                               tRNA-Tyr COX1
Pigeon (GU908131)  ATAAGAAGAGGAATTTAACCTCTGTAAAAAGGACTACAGCCTAACGCTTCATACACTCAGCCATCTTACCTGTGACCCTA 5389
JQ692598           .................................................A.A.......................T.A.. 5258
BMNH1149           .................................................A.A.......................T.A.. 5418
BMNH1389           .................................................A.A.......................T.A.. 5380

                   COX1
Pigeon (GU908131)  ATCAATCGATGATTATTCTCTACTAACCACAAAGACATCGGCACCCTATACCTAATCTTCGGCGCATGGGCCGGCATAGT 5469
JQ692598           ............C.......C........T........T..T.....G..............T.....A.....T..... 5338
BMNH1149           ............C.......C........T........T..T.....G..............T.....A.....T..... 5498
BMNH1389           ............C.......C........T........T..T.....G..............T.....A.....T..... 5460

                   COX1
Pigeon (GU908131)  TGGCACCGCACTTAGCCTCCTCATCCGAGCAGAACTGGGACAACCCGGTACCCTCCTAGGAGATGACCAGATCTATAATG 5549
JQ692598           ........................T..T........A........T.......................A.....C.... 5418
BMNH1149           ........................T..T........A........T.......................A.....C.... 5578
BMNH1389           ........................T..T........A........T.......................A.....C.... 5540
```

```
                    COX1
Pigeon (GU908131)   TAATCGTCACAGCTCATGCCTTCGTAATAATCTTCTTTATAGTTATACCAATCATAATTGGGGGCTTTGGAAACTGATTA 5629
JQ692598            ....T..T.....C..............G........C.....C..G...........C..A.....C............ 5498
BMNH1149            ....T..T.....C..............G........C.....C..G...........C..A.....C............ 5658
BMNH1389            ....T..T.....C..............G........C.....C..G...........C..A.....C............ 5620
                    COX1
Pigeon (GU908131)   GTTCCCCTCATAATTGGTGCCCCCGACATAGCATTCCCCCGGATGAACAACATAAGCTTCTGGCTCCTACCCCCATCCTT 5709
JQ692598            ..C...........C....................T..A..A..A.................A..A..C........... 5578
BMNH1149            ..C...........C....................T..A..A..A.................A..A..C........... 5738
BMNH1389            ..C...........C....................T..A..A..A.................A..A..C........... 5700
                    COX1
Pigeon (GU908131)   CCTTCTACTTCTAGCCTCCTCCACAGTCGAAGCTGGTGCAGGAACAGGATGAACCGTCTACCCTCCCCTAGCTGGCAACC 5789
JQ692598            T.....C..................................................T..............C......T 5658
BMNH1149            T.....C..................................................T..............C......T 5818
BMNH1389            T.....C..................................................T..............C......T 5780
                    COX1
Pigeon (GU908131)   TAGCTCACGCCGGAGCCTCTGTAGACCTTGCCATCTTCTCCCTTCACCTTGCTGGTATCTCCTCTATTCTAGGGGCCATC 5869
JQ692598            ....C.....G........C....................A..C............G.......C..C............ 5738
BMNH1149            ....C.....G........C....................A..C............G.......C..C............ 5898
BMNH1389            ....C.....G........C....................A..C............G.......C..C............ 5860
                    COX1
Pigeon (GU908131)   AACTTCATCACAACTGCCATTAACATAAAACCCCCAGCCCTCTCACAATACCAAACCCCACTATTCGTCTGATCAGTCCT 5949
JQ692598            .....T........C........T...........C.....T..............T..C........A.....G..... 5818
BMNH1149            .....T........C........T...........C.....T..............T..C........A.....G..... 5978
BMNH1389            .....T........C........T...........C.....T..............T..C........A.....G..... 5940
                    COX1
Pigeon (GU908131)   CATCACTGCCGTCCTCCTTTTACTATCCCTCCCAGTACTTGCCGCCGGCATCACAATACTGCTCACAGACCGAAACCTAA 6029
JQ692598            ...T..C............C.C..............C........T............T.A........T........C. 5898
BMNH1149            ...T..C............C.C..............C........T............T.A........T........C. 6058
BMNH1389            ...T..C............C.C..............C........T............T.A........T........C. 6020
                    COX1
Pigeon (GU908131)   ACACTACCTTCTTTGATCCTGCTGGTGGAGGGGACCCAGTACTATACCAACATCTCTTCTGATTCTTTGGTCATCCTGAA 6109
JQ692598            ....C........C..C..............T..T........G..........................C..C..C... 5978
BMNH1149            ....C........C..C..............T..T........G..........................C..C..C... 6138
BMNH1389            ....C........C..C..............C..T........G..........................C..C..C... 6100
                    COX1
Pigeon (GU908131)   GTCTATATCCTAATTTTACCAGGATTCGGGATTATCTCCCACGTAGTGGCCTACTACGCAGGTAAAAAAGAACCCTTCGG 6189
JQ692598            ..T.................G..T.....A..C........T.....A........T..C.....G...........T.. 6058
BMNH1149            ..T.................G..T.....A..C........T.....A........T..C.....G...........T.. 6218
BMNH1389            ..T.................G..T.....A..C........T.....A........T..C.....G...........T.. 6180
```

```
                   COX1
Pigeon (GU908131)  CTACATAGGCATAGTATGAGCCATGCTATCTATTGGCTTCCTAGGCTTTATCGTTTGAGCTCACCATATATTTACAGTAG 6269
JQ692598           ...T..G.....G...........A...........A.................C...........C.....C....... 6138
BMNH1149           ...T..G.....G...........A...........A.................C...........C.....C....... 6298
BMNH1389           ...T..G.....G...........A...........A.................C...........C.....C....... 6260
                   COX1
Pigeon (GU908131)  GCATGGACGTAGACACCCGAGCATACTTCACATCAGCCACTATAATCATTGCCATCCCAACGGGCATTAAAGTCTTCAGC 6349
JQ692598           ....A..T.........................................C.................C............ 6218
BMNH1149           ....A..T.........................................C.................C............ 6378
BMNH1389           ....A..T.........................................C.................C............ 6340
                   COX1
Pigeon (GU908131)  TGACTAGCCACTCTCCATGGCGGGACTATCAAATGAGACCCCCCAATATTATGAGCCCTAGGCTTCATCTTCCTCTTCAC 6429
JQ692598           ..GT.......A..T..C..G........T........T..T..C...C....G.....G.................T.. 6298
BMNH1149           ..GT.......A..T..C..G........T........T..T..C...C....G.....G.................T.. 6458
BMNH1389           ..GT.......A..T..C..G........T........T..T..C...C....G.....G.................T.. 6420
                   COX1
Pigeon (GU908131)  CATTGGAGGCCTTACAGGAATTGTCCTAGCAAACTCCTCCCTAGACATTGCTCTACACGACACATACTACGTAGTCGCTC 6509
JQ692598           ............A.....G.....A...........T..............C..C.....T..G...........T..C. 6378
BMNH1149           ............A.....G.....A...........T..............C..C.....T..G...........T..C. 6538
BMNH1389           ............A.....G.....A...........T..............C..C.....T..G...........T..C. 6500
                   COX1
Pigeon (GU908131)  ACTTCCACTATGTCCTCTCAATAGGAGCTGTCTTCGCTATTCTAGCAGGATTCACTCATTGATTCCCACTATTCACAGGA 6589
JQ692598           ..........C..A..T..............A.....C..C...........T.....C..............T...... 6458
BMNH1149           ..........C..A..T..............A.....C..C...........T.....C..............T...... 6618
BMNH1389           ..........C..A..T..............A.....C..C...........T.....C..............T...... 6580
                   COX1
Pigeon (GU908131)  TATACCCTCCACCCCACATGAGCTAAAGCCCATTTCGGAGTAATATTCACCGGCGTCAACTTAACATTCTTCCCCCAACA 6669
JQ692598           ........AA.T....................C........T..................C................... 6538
BMNH1149           ........AA.T....................C........T..................C................... 6698
BMNH1389           ........AA.T....................C........T..................C................... 6660
                   COX1
Pigeon (GU908131)  CTTCCTCGGCCTTGCTGGCATACCACGACGATACTCAGACTACCCAGACGCCTACACCCTGTGAAACACTATCTCCTCTA 6749
JQ692598           ............C........G........G..............G.....A........A.................A. 6618
BMNH1149           ............C........G........G..............G.....A........A.................A. 6778
BMNH1389           ............C........G........G..............G.....A........A.................A. 6740
                   COX1
Pigeon (GU908131)  TCGGATCATTAATCTCAATAACAGCCGTAATCATACTAATATTCATCATCTGAGAAGCCTTCGCATCAAAACGCAAAGTA 6829
JQ692598           .T..G..TC..........................T.......T..............T...........G..T...... 6698
BMNH1149           .T..G..TC..........................T.......T..............T...........G..T...... 6858
BMNH1389           .T..G..TC..........................T.......T..............T...........G..T...A.. 6820
```

```
COX1
Pigeon (GU908131) CTACAACCAGAACTTACCTCCACCAACATCGAGTGAATCCACGGCTGCCCACCTCCATACCACACCTTCGAGGAACCAGC 6909
JQ692598          ..G...........A..............T..A....................C.................A........ 6778
BMNH1149          ..G...........A..............T..A....................C.................A........ 6938
BMNH1389          ..G...........A..............T..A....................C.................A........ 6900

COX1                                                                            tRNA-Ser
Pigeon (GU908131) CTTCGTCCAAGTACAAGAAAGGAAGGAATCGAACCCTCGTACGCTGGTTTCAAGCCAACCGCATCAAACCACTTATGCTT 6989
JQ692598          ............C..............G.................................................... 6858
BMNH1149          ............C..............G.................................................... 7018
BMNH1389          ............C..............G.................................................... 6980

tRNA-Ser   tRNA-Asp
Pigeon (GU908131) CTTTCTTATGAGACGTTAGTAAACCTATTACATAGCCTTGTCAAGACTAAATCACAGGTGAAAATCCTGTACTTCTCACT 7069
JQ692598          ...............................................................................C 6938
BMNH1149          ...............................................................................C 7098
BMNH1389          ...............................................................................C 7060

COX2
Pigeon (GU908131) ATGGCTAACCACTCACAATTCGGATTTCAAGATGCCTCATCCCCTATCATAGAAGAACTCGTTGAATTCCACGACCACGC 7149
JQ692598          .....C...................................T..C..............T........T........T.. 7018
BMNH1149          .....C...................................T..C..............T........T........T.. 7178
BMNH1389          .....C...................................T..C..............T........T........T.. 7140

COX2
Pigeon (GU908131) TCTAATAGTTGCGCTAGCAATCTGCAGCCTAGTTCTTTACCTTTTAACACTTATACTAATAGAAAAGCTCTCCTCAAACA 7229
JQ692598          C..G.....C..TT..........T........C..C..T...C...T...C..............A..A.......... 7098
BMNH1149          C..G.....C..TT..........T........C..C..T...C...T...C..............A..A.......... 7258
BMNH1389          C..G.....C..TT..........T........C..C..T...C...T...C..............A..A.......... 7220

COX2
Pigeon (GU908131) CTGTAGATGCCCAAGAAGTCGAGCTAATCTGAACAATCCTACCAGCTATTGTCCTCATCCTACTCGCCCTTCCATCACTA 7309
JQ692598          ......................A.................G.....C..C.....T...T..........C.....C... 7178
BMNH1149          ......................A.................G.....C..C.....T...T..........C.....C... 7338
BMNH1389          ......................A.................G.....C..C.....T...T..........C.....C... 7300

COX2
Pigeon (GU908131) CAAATCCTCTACATAATAGACGAAATTGACGAACCAGACCTAACCCTAAAAGCCATCGGACATCAGTGATACTGATCCTA 7389
JQ692598          ........T.....G..G........C............T.............T.....T.....A..G........... 7258
BMNH1149          ........T.....G..G........C............T.............T.....T.....A..G........... 7418
BMNH1389          ........T.....G..G........C............T.............T.....T.....A..G........... 7380
```

	COX2	
Pigeon (GU908131)	CGAATATACAGACTTCAAAGACCTAACATTCGACTCCTACATAATCCCCACAGCAGAGCTCCCACCAGGACACTTCCGAC	7469
JQ692598	...G.....G..T............G....T.................G...A....A..............T.......	7338
BMNH1149	...G.....G..T............G....T.................G...A....A..............T.......	7498
BMNH1389	...G.....G..T............G....T.................G...A....A..............T.......	7460
	COX2	
Pigeon (GU908131)	TACTAGAAGTAGACCACCGCGTTGTTGTTCCTATAGAATCTCCAATTCGCATCATCGTCACTGCCGACGACGTCCTACAC	7549
JQ692598	.T..............T..A...A....A...........C........T........T..C..T.....T.........	7418
BMNH1149	.T..............T..A...A....A...........C........T........T..C..T.....T.........	7578
BMNH1389	.T..............T..A...A....A...........C........T........T..C..T.....T.........	7540
	COX2	
Pigeon (GU908131)	TCCTGAGCAGTCCCCTCCCTCGGAGTAAAAACCGATGCAATTCCAGGACGACTAAACCAAACATCATTCATTACCGCCCG	7629
JQ692598	..............A.....T..............C.....C..T..G.....G.................C...A....	7498
BMNH1149	..............A.....T..............C.....C..T..G.....G.................C...A....	7658
BMNH1389	..............A.....T..............C.....C..T..G.....G.................C...A....	7620
	COX2	
Pigeon (GU908131)	ACCAGGAATCTTCTATGGCCAATGCTCAGAAATCTGTGGGGCTAACCATAGTTATATGCCAATTGTAGTAGAATCGACCC	7709
JQ692598	...C...G.G........A..G..............C..A.....T..C..C..C..A.....C...........A..TT	7578
BMNH1149	...C...G.G........A..G..............C..A.....T..C..C..C..A.....C...........A..TT	7738
BMNH1389	...C...G.G........A..G..............C..A.....T..C..C..C..A.....C...........A..TT	7700
	COX2 … tRNA-Lys	
Pigeon (GU908131)	CACTCGCCCACTTCGAATCCTGATCCACACTATTATCCTCATAA T CATTAAGAAGCTATATGCTAGCGCTAGCCTTTTAA	7789
JQ692598	....TA.A........G.......................T...................CA.C................	7658
BMNH1149	....TA.A........G.......................T...................CA.C................	7818
BMNH1389	....TA.A........G.......................T...................CA.C................	7780
	tRNA-Lys … ATP8	
Pigeon (GU908131)	GCTAGAGAAAGAGGATTGCCCGCCCCTCCTTAATGA C ATGCCCCAGCTCAATCCAAACCCATGATTCTTCATCATACTCC	7869
JQ692598	...............CCA..TA..............T........A..A..C..............T..T........T.	7738
BMNH1149	...............CCA..TA..............T........A..A..C..............T..T........T.	7898
BMNH1389	...............CCA..TA..............T........A..A..C..............T..T........T.	7860
	ATP8	
Pigeon (GU908131)	TATCATGATTAACCTTCTCCTTAATTATCCAACCCAAGCTCCTATCTTTCACCCCCACCAACCCCCCATCTAGCAAAATC	7949
JQ692598	.G......C....A......C....C.....G.....A........A..T.TT.....T...........C.A.....C.	7818
BMNH1149	.G......C....A......C....C.....G.....A........A..T.TT.....T...........C.A.....C.	7978
BMNH1389	.G......C....A......C....C.....G.....A........A..T.TT.....T...........C.A.....C.	7940

```
                   ATP8                                          ATP6
Pigeon (GU908131)  CTAACCACCACAAAATCAACACCCTGAACCTGACCATGAACTTAAGCTTCTTTGATCAATTTACAAGCCCGTACCTTCTA 8029
JQ692598           ........TGT.........................................C..C.....C........A.G...C... 7898
BMNH1149           ........TGT.........................................C..C.....C........A.G...C... 8058
BMNH1389           ........TGT.........................................C..C.....C........A.G...C... 8020
                   ATP6
Pigeon (GU908131)  GGAATTCCACTGATCCTTATCTCAATACTATTCCCTGCACTACTCCTTCCATCCCCTAACAACCGTTGAATTACTAACCG 8109
JQ692598           .....C...........AC..........G.....CA.C.....A..C..G..A..C.....T........C.....T.. 7978
BMNH1149           .....C...........AC..........G.....CA.C.....A..C..G..A..C.....T........C.....T.. 8138
BMNH1389           .....C...........AC..........G.....CA.C.....A..C..G..A..C.....T........C.....T.. 8100
                   ATP6
Pigeon (GU908131)  CCTCTCTACTCTCCAACTCTGACTCTTTCACCTGATTACAAAACAACTAATAATCCCATTAAACAAAAACGGCCACAAAT 8189
JQ692598           ...A..C..C........T...T..C....T..A..C.....G.....C.........C.G..T...........T.... 8058
BMNH1149           ...A..C..C........T...T..C....T..A..C.....G.....C.........C.G..T...........T.... 8218
BMNH1389           ...A..C..C........T...T..C....T..A..C.....G.....C.........C.G..T...........T.... 8180
                   ATP6
Pigeon (GU908131)  GAGCCTTACTACTAACCTCCCTAATAACCCTCCTACTCACAATTAACCTCTTAGGTCTTCTACCATATACATTCACCCCA 8269
JQ692598           .....C....CT..............GTT..AT.....T...................C........C............ 8138
BMNH1149           .....C....CT..............GTT..AT.....T...................C........C............ 8298
BMNH1389           .....C....CT..............GTT..AT.....T...................C........C............ 8260
                   ATP6
Pigeon (GU908131)  ACTACCCAGCTATCGATAAACATAGCCCTAGCATTCCCACTCTGACTAGCCACCCTCCTCACAGGCCTACGCAATCAACC 8349
JQ692598           ..C......T....A...........T.....G.....G..............T..T...........G.....C..... 8218
BMNH1149           ..C......T....A...........T.....G.....G..............T..T...........G.....C..... 8378
BMNH1389           ..C......T....A...........T.....G.....G..............T..T...........G.....C..... 8340
                   ATP6
Pigeon (GU908131)  CTCAATTGCTCTAGGTCACCTTTTACCCGAAGGCACACCTACCCCACTAATCCCTGCCCTGATCATGATCGAAACTACTA 8429
JQ692598           .........C.....C......C...................A...........A.....A...C.A..T........C. 8298
BMNH1149           .........C.....C......C...................A...........A.....A...C.A..T........C. 8458
BMNH1389           .........C.....C......C...................A...........A.....A...C.A..T........C. 8420
                   ATP6
Pigeon (GU908131)  GTCTATTAATTCGCCCATTAGCCCTAGGAGTCCGTCTCACAGCTAATCTCACAGCAGGTCACCTCCTCATCCAGCTTATC 8509
JQ692598           .C................................A...........C..T........C..T...........A..C... 8378
BMNH1149           .C................................A...........C..T........C..T...........A..C... 8538
BMNH1389           .C................................A...........C..T........C..T...........A..C... 8500
```

ATP6

Pigeon (GU908131)	TCTACAGCCACAACCGCCCTCCTCCCCATCATACCGACAATTTCACTCCTAACAGCATTAATCTTATTCCTCTTAACCAT	8589
JQ692598	.....G..T..............T..A..T......G..G.C...G............C....C........C.G.....	8458
BMNH1149	.....G..T..............T..A..T......G..G.C...G............C....C........C.G.....	8618
BMNH1389	.....G..T..............T..A..T......G..G.C...G............C....C........C.G.....	8580

ATP6 / COX3

Pigeon (GU908131)	CCTAGAGGTAGCAGTAGCCATAATCCAAGCGTACGTCTTCGTCCTACTCCTAAGCCTCTACTTACAAGAAAACATCTAAT	8669
JQ692598	.T....A...........T...........C..................T....T.................T..T....	8538
BMNH1149	.T....A...........T...........C..................T....T.................T..T....	8698
BMNH1389	.T....A...........T...........C..................T....T.................T..T....	8660

COX3

Pigeon (GU908131)	GGCCCACCAAGCCCACTCCTACCACATAGTAGACCCAAGCCCCTGACCCATTCTAGGAGCAACAGCCGCCCTACTCACCA	8749
JQ692598	...T........T......................................C..............T........T..T.	8618
BMNH1149	...T........T......................................C..............T........T..T.	8778
BMNH1389	...T........T......................................C..............T........T..T.	8740

COX3

Pigeon (GU908131)	CCTCAGGACTAATCATATGATTCCACTTCAACACATCATACCTCCTGGCCTTAGGGCTCCTATCCATACTCTTAGTTATA	8829
JQ692598	......................T....A......C...C.......A..TC....A...A.C..T..G..T.....C...	8698
BMNH1149	......................T....A......C...C.......A..TC....A...A.C..T..G..T.....C...	8858
BMNH1389	......................T....A......C...C.......A..TC....A...A.C..T..G..T.....C...	8820

COX3

Pigeon (GU908131)	CTGCAATGATGACGCGACATTGTACGAGAAAGTACATTCCAAGGTCACCACACCCCCACCGTACAAAAAGGCCTACGATA	8909
JQ692598	T.A...........T...........T.................C.............................T.....	8778
BMNH1149	T.A...........T...........T.................C.............................T.....	8938
BMNH1389	T.A...........T...........T.................C.............................T.....	8900

COX3

Pigeon (GU908131)	CGGAATAATCCTATTCATCACATCAGAAGCATTCTTCTTCCTCGGCTTCTTCTGAGCCTTCTTCCACTCTAGCCTAGCTC	8989
JQ692598	....................................T..T..T........T.....A...........A.....G..C.	8858
BMNH1149	....................................T..T..T........T.....A...........A.....G..C.	9018
BMNH1389	....................................T..T..T........T.....A...........A.....G..C.	8980

COX3

Pigeon (GU908131)	CCACCCCAGAGCTAGGTGGCCAATGACCTCCCACTGGAATCAACCCGCTCAACCCCCTAGAAGTCCCCCTACTAAACACA	9069
JQ692598	.A........A........A........C..A..G.................T...........T..T............	8938
BMNH1149	.A........A........A........C..A..G.................T...........T..T............	9098
BMNH1389	.A..T.....A........A........C..A..G.................T...........T..T............	9060

Sequence	COX3	
Pigeon (GU908131)	GCTATCCTACTCGCTTCCGGTGTTACTGTTACATGAGCCCACCACAGCATCACAGAAAGCAACCGAAAACAAGCCATCCA	9149
JQ692598	........C...........C........C........A..................G.............G........	9018
BMNH1149	........C...........C........C........A..................G.............G........	9178
BMNH1389	........C...........C........C........A..................G.............G........	9140

Sequence	COX3	
Pigeon (GU908131)	AGCACTTGCCCTAACCATCCTACTAGGGTTCTACTTTACAGCTCTCCAAGCCACAGAATACTATGAAGCGCCCTTCTCCA	9229
JQ692598	......CA..........T........A........C.....C....................C.....A..........	9098
BMNH1149	......CA..........T........A........C.....C....................C.....A..........	9258
BMNH1389	......CA..........T........A........C.....C....................C.....A..........	9220

Sequence	COX3	
Pigeon (GU908131)	TCGCCGATGGAGTATATGGTTCAACTTTTTTCGTCGCCACAGGATTCCATGGCCTCCATGTCATCATTGGATCCTCATTC	9309
JQ692598	.......C..G........C.....C...........T...........C.....G..C........C............	9178
BMNH1149	.......C..G........C.....C...........T...........C.....G..C........C............	9338
BMNH1389	.......C..G........C.....C...........T...........C.....G..C........C............	9300

Sequence	COX3	
Pigeon (GU908131)	CTTTCAGTCTGCCTCCTACGACTAATCAAATTCCACTTTACATCTAACCATCACTTCGGATTTGAAGCAGCAGCCTGATA	9389
JQ692598	..C..............G........T...........C.....A.G...C.................C...........	9258
BMNH1149	..C..............G........T...........C.....A.G...C.................C...........	9418
BMNH1389	..C..............G........T...........C.....A.G...C.................C...........	9380

Sequence	COX3 / tRNA-Gly	
Pigeon (GU908131)	TTGACACTTCGTAGACGTTATCTGATTATTCCTCTACATGACCATCTACTGATGAGGATCAT / GCTCTTCTAGTATACTGA	9469
JQ692598	C.................C....................A..T..T................................A.	9338
BMNH1149	C.................C....................A..T..T................................A.	9498
BMNH1389	C.................C....................A..T..T................................A.	9460

Sequence	tRNA-Gly / ND3	
Pigeon (GU908131)	TTACAATCGACTTCCAATCCTTAAAATCTGGTGGAACCCCAGAGAAGAGCA / ATTAACATAATCACATTCATACTCACCTT	9549
JQ692598	.................................T...................C........T..T.....G......C.	9418
BMNH1149	.................................T...................C........T..T.....G......C.	9578
BMNH1389	.................................T...................C........T..T.....G......C.	9540

Sequence	ND3	
Pigeon (GU908131)	ATCCCTTATCCTATCCATCCTCTTAACCACATTAAACTTTTGACTTGCCCAAATAAACCCAGACCTAGAAAAACTATCCC	9629
JQ692598	......CGC.....GT..T..TC....T........T..C.....C..................................	9498
BMNH1149	......CGC.....GT..T..TC....T........T..C.....C..................................	9658
BMNH1389	......CGC.....GT..T..TC....T........T..C.....C..................................	9620

Sequence	ND3	
Pigeon (GU908131)	CGTACGAATGCGGCTTCGACCCCTTGGGATCCGCTCGACTCCCATTTTCAATCCGATTCTTCCTCAGTAGCCATCCTCTT	9709
JQ692598	.A..............T.....TC.C.....T...........T..C...........................T..T..	9578
BMNH1149	.A..............T.....TC.C.....T...........T..C...........................T..T..	9738
BMNH1389	.A..............T.....TC.C.....T...........T..C...........................T..T..	9700

```
                    ND3
Pigeon (GU908131)   CCTCCTCTTCGACCTAGAAATTGCCCTCCTCCTGCCCCTCCCATGAGCCAGCCAACTTCAATCCCCTGTCACCACACTAA 9789
JQ692598            ...T..T..............C...........C.....T........T........C........CACT........C. 9658
BMNH1149            ...T..T..............C...........C.....T........T........C........CACT........C. 9818
BMNH1389            ...T..T..............C...........C.....T........T........C........CACT........C. 9780

                    ND3
Pigeon (GU908131)   CTTGAGCCTCCACCCTTATTCTCCTACTAACACTAGGACTAATCTATGAATGGGTGCAAGGAGGACTAGAATGAGCAGAA 9869
JQ692598            TC........T.....C.................G...T.G.....C..G..AA...........T.............. 9738
BMNH1149            TC........T.....C.................G...T.G.....C..G..AA...........T.............. 9898
BMNH1389            TC........T.....C.................G...T.G.....C..G..AA...........T.............. 9860

                    ND3  tRNA-Arg                                                            ND4L
Pigeon (GU908131)   TAATAGAAAGTTAGTCTAACTAAGACAGTTGATTTCGGCTCAACAGACCATAGCTCAACCCTATGACTTTCTTAATGTCA 9949
JQ692598            ...C................C...................................G................C.....G 9818
BMNH1149            ...C................C...................................G................C.....G 9978
BMNH1389            ...C................C...................................G................C.....G 9940

                    ND4L
Pigeon (GU908131)   CTCCTCCACCTGAGCTTCTACTCCGCCTTCACCTTAAGTGGCCTAGGATTAGCCTTCCACCGAACCCACCTAATCTCTGC 10029
JQ692598            .CT..A.....T.....................C....CA..................................T..C.. 9898
BMNH1149            .CT..A.....T.....................C....CA..................................T..C.. 10058
BMNH1389            .CT..A.....T.....................C....CA..................................T..C.. 10020

                    ND4L
Pigeon (GU908131)   CCTACTATGTCTAGAGAGCATAATACTATCTATATACCTCGCCCTATCGATCTGACCTATCGAAAACCAAGCAACGTCAT 10109
JQ692598            T........CT....A........G.....C...........T.....A..T.......................A..C. 9978
BMNH1149            T........CT....A........G.....C...........T.....A..T.......................A..C. 10138
BMNH1389            T........CT....A........G.....C...........T.....A..T.......................A..C. 10100

                    ND4L
Pigeon (GU908131)   TCACCCTGACACCTGTACTCATACTAGCATTCTCAGCATGCGAAGCAGGAGCCGGCCTAGCCATGCTAGTAGCCTCAACA 10189
JQ692598            C......AGT...C..C..T.....G.................G....................A...........T... 10058
BMNH1149            C......AGT...C..C..T.....G.................G....................A...........T... 10218
BMNH1389            C......AGT...C..C..T.....G.................G....................A...........T... 10180

                    ND4L                                        ND4
Pigeon (GU908131)   CGAACTCATGGTTCAGATCACTTACACAATCTAAACCTCCTACAATGCTAAAAATCATCCTCCCCACAATCATACTCATC 10269
JQ692598            ........C..C.....C...........C........T................T..T.....T..............A 10138
BMNH1149            ........C..C.....C...........C........T................T..T.....T..............A 10298
BMNH1389            ........C..C.....C...........C........T................T..T.....T..............A 10260
```

```
                    ND4
Pigeon (GU908131)   CCCACAGCCCTCCTATCACCCAAAAAATTCTTATGAACTAACACCACCACGTACAGCCTACTAATCGCCACCCTAAGCCT 10349
JQ692598            ..TGT.A..........C........G...C.G.....A...........A..T.....C.....T.....T........ 10218
BMNH1149            ..TGT.A..........C........G...C.G.....A...........A..T.....C.....T.....T........ 10378
BMNH1389            ..TGT.A..........C........G...C.G.....A...........A..T.....C.....T.....T........ 10340
                    ND4
Pigeon (GU908131)   CCAGTGACTACTCCCCTCGCACTACCCACACAAAAACATAACCCCATGAACTGGCATCGACCAGATCTCATCCCCACTAC 10429
JQ692598            T..A...........A..AT...T...T.......................C.....T.....A........T.....GT 10298
BMNH1149            T..A...........A..AT...T...T.......................C.....T.....A........T.....GT 10458
BMNH1389            T..A...........A..AT...T...T.......................C.....T.....A........T.....GT 10420
                    ND4
Pigeon (GU908131)   TAGTCTTATCCTGCTGACTACTACCCCTCATAATCATAGCAAGCCAAAATCACCTTCAACACGAGCCTCCCACACGAAAG 10509
JQ692598            .....C....A........C.....T.......................C.....A........A..CA..........A 10378
BMNH1149            .....C....A........C.....T.......................C.....A........A..CA..........A 10538
BMNH1389            .....C....A........C.....T.......................C.....A........A..CA..........A 10500
                    ND4
Pigeon (GU908131)   CGGATCTTCATTACAACGCTAATCACAATCCAACCCTTCATCCTACTAGCCTTCGCATCCACTGAACTAATACTGTTCTA 10589
JQ692598            ..A............G.A...G.T.....T.....T..T...A.C.........T..........G......T.A..... 10458
BMNH1149            ..A............G.A...G.T.....T.....T..T...A.C.........T..........G......T.A..... 10618
BMNH1389            ..A............G.A...G.T.....T.....T..T...A.C.........T..........G......T.A..... 10580
                    ND4
Pigeon (GU908131)   CATCTCATTCGAAGCAACCCTAATCCCTACACTAATTCTCATCACACGATGGGGAAACCAACCTGAACGCCTAAGCGCTG 10669
JQ692598            .........T....................T.....C..............A.................AT......... 10538
BMNH1149            .........T....................T.....C..............A.................AT......... 10698
BMNH1389            .........T....................T.....C..............A.................AT......... 10660
                    ND4
Pigeon (GU908131)   GCATTTACTTATTATTCTATACCCTCATCAGCTCCCTCCCACTACTAGTTGCAATACTATACCTACACATACAAATCGGC 10749
JQ692598            ...................C.................T.....G.......T...G..C...T....T.....G...... 10618
BMNH1149            ...................C.................T.....G.......T...G..C...T....T.....G...... 10778
BMNH1389            ...................C.................T.....G.......T...G..C...T....T.....G...... 10740
                    ND4
Pigeon (GU908131)   ACCCTACACCTCACAATACTCAAACTAACCCACCCTCCCCTTAACAACTCCTGAACTGGCCTCCTATCTAGCCTAGCACT 10829
JQ692598            ...........T.....G..T......G........G....C................AT........AG....T..... 10698
BMNH1149            ...........T.....G..T......G........G....C................AT........AG....T..... 10858
BMNH1389            ...........T.....G..T......G........G....C................AT........AG....T..... 10820
                    ND4
Pigeon (GU908131)   ACTAATAGCATTCATAGTAAAAGCACCCTTATACGGCCTGCACCTATGACTACCCAAAGCCCACGTCGAAGCACCAATCG 10909
JQ692598            ............T..............AC....T..T..T...........G............................ 10778
BMNH1149            ............T..............AC....T..T..T...........G............................ 10938
BMNH1389            ............T..............AC....T..T..T...........G............................ 10900
```

	ND4	
Pigeon (GU908131)	CAGGATCAATGTTATTCGCAGCCCTACTTCTTGAGCTAGGCGGCTATGGCATCATACGAGTTACCCTCCTAATAAGCCCC	10989
JQ692598	....G.....AC..C.T..C........C...A.AT....G..A.....T........TA.C............G....A	10858
BMNH1149	....G.....AC..C.T..C........C...A.AT....G..A.....T........TA.C............G....A	11018
BMNH1389	....G.....AC..C.T..C........C...A.AT....G..A.....T........TA.C............G....A	10980
	ND4	
Pigeon (GU908131)	ACCTCCAACCACCTATACTACCCATTCATTACCTTAGCCCTATGGGGCGCCCTAATAACTAGCTCAATCTGCTTGCGCCA	11069
JQ692598	.T.........T...C...................G........A......T....................C.A..T..	10938
BMNH1149	.T.........T...C...................G........A......T....................C.A..T..	11098
BMNH1389	.T.........T...C...................G........A......T....................C.A..T..	11060
	ND4	
Pigeon (GU908131)	AACGGACCTAAAGTCCCTCATTGCCTACTCTTCCGTTAGCCATATAGGCCTAGTCATCGCTGCAAGCATAATTCAAACCC	11149
JQ692598	G..C.....C...........C........C..T..C.....C..............T..C...................	11018
BMNH1149	G..C.....C...........C........C..T..C.....C..............T..C...................	11178
BMNH1389	G..C.....C...........C........C..T..C.....C..............T..C...................	11140
	ND4	
Pigeon (GU908131)	ACTGAGCATTCTCAGGCGCAATACTCCTTATAATCTCACACGGACTAACCTCTTCCATACTATTCTGCTTAGCTAACACA	11229
JQ692598	.......................A.............C...........T..C....................C......	11098
BMNH1149	.......................A.............C...........T..C....................C......	11258
BMNH1389	.......................A.............C...........T..C....................C......	11220
	ND4	
Pigeon (GU908131)	AACTACGAGCGAACACGTAGCCGAATCTTGATCCTAACACGAGGCCTACAACCCCTCCTACCCCTAATAGCCACCTGATG	11309
JQ692598	.....T..A.......A..........C.AC................T..G.....AT.G....................	11178
BMNH1149	.....T..A.......A..........C.AC................T..G.....AT.G....................	11338
BMNH1389	.....T..A.......A..........C.AC................T..G.....AT.G....................	11300
	ND4	
Pigeon (GU908131)	ACTCCTAGCCAACCTCACAAACATAGCCCTCCCTCCAACAACAAATCTTATAGCAGAACTAACCATTATAATCGCACTGT	11389
JQ692598	G..TT....T.....A...........T.....C...........CT.A..............T..............A.	11258
BMNH1149	G..TT....T.....A...........T.....C...........CT.A..............T..............A.	11418
BMNH1389	G..TT....T.....A...........T.....C...........CT.A..............T..............A.	11380
	ND4	
Pigeon (GU908131)	TCAACTGGTCCACTCCAACTATCATTTTAACCGGAATCGCAACCCTACTAACCGCTTCATATACCCTATTCATACTCCTA	11469
JQ692598	.......A..TG....C..A..T...C.........CA........G.....T..C.....C.......A......A...	11338
BMNH1149	.......A..TG....C..A..T...C.........CA........G.....T..C.....C.......A......A...	11498
BMNH1389	.......A..TG....C..A..T...C.........CA........G.....T..C.....C.......A......A...	11460
	ND4	
Pigeon (GU908131)	ATAACCCAACGAGGAACCATACCCACTCACATCACATCCATTCAGAACTCAACCACACGAGAGCATCTCCTAATATCCCT	11549
JQ692598	..................C.........................A....................C.....C........	11418
BMNH1149	..................C.........................A....................C.....C........	11578
BMNH1389	..................C.........................A....................C.....C........	11540

```
                   ND4                                                              tRNA-His (from position 64)
Pigeon (GU908131)  CCACATCCTCCCCATATTACTCCTCATCATAAAACCTAACCTAATCTCAGGAATCCTCGCATGCAAGTATAGTTTAACCC 11629
JQ692598           .........T......C...........T........G....C.....C...GC....T..................... 11498
BMNH1149           .........T......C...........T........G....C.....C...GC....T..................... 11658
BMNH1389           .........T......C...........T........G....C.....C...GC....T..................... 11620

                   tRNA-His                                           tRNA-Ser (from position 52)
Pigeon (GU908131)  AAACATTAGACTGTGATCCTAAAAATAGAAGTTAGACCCTTCTTACCTGCCGAGGGGAGGTTCAACCAACAAGAACTGCT 11709
JQ692598           ..................................A............................................. 11578
BMNH1149           ..................................A............................................. 11738
BMNH1389           ..................................A............................................. 11700

                   tRNA-Ser                            tRNA-Leu (from position 37)
Pigeon (GU908131)  AATTCCTGTATCTGAGTCTAAAACCTCAGCCCCCTTACTTTTAAAGGATAATAGCTAATCCACTGGTCTTAGGAGCCATT 11789
JQ692598           ......................................................-......................... 11657
BMNH1149           ......................................................-......................... 11817
BMNH1389           ......................................................-......................... 11779

                   tRNA-Leu                    ND5 (from position 29)
Pigeon (GU908131)  CATCTTGGTGCAAGTCCAAGTAAAAGTAGTGGAACATGCCCTACTCCCTAACACCTCCATCCTCCTAACATTTGCAATTA 11869
JQ692598           ...................................C...........TC.................G...C..A....C. 11737
BMNH1149           ...................................C...........TC.................G...C..A....C. 11897
BMNH1389           ...................................C...........TC.................G...C..A....C. 11859

                   ND5
Pigeon (GU908131)  TTCTTATACCCATCCTACTGCCACTCATATCAAAAAACTTATCAAACTCCCCAGCCACCATTACACACACTGTCAAAATC 11949
JQ692598           .C..C.CC...G....C..T..........T.........TCA....A.....A.......C................CT 11817
BMNH1149           .C..C.CC...G....C..T..........T.........TCA....A.....A.......C................CT 11977
BMNH1389           .C..C.CC...G....C..T..........T.........TCA....A.....A.......C................CT 11939

                   ND5
Pigeon (GU908131)  GCATTTCTAACAAGCCTAGTGCCAATGACACTATTCATGTACCTAGGCCCAGAAAGCATCACCTCCCACCTAGAATGAAA 12029
JQ692598           .....CA.C..G........A.....A.....C.....A...TC....T....G.......T......T..G........ 11897
BMNH1149           .....CA.C..G........A.....AT....C.....A...TC....T....G.......T......T..G........ 12057
BMNH1389           .....CA.C..G........A.....A.....C.....A...TC....T....G.......T......T..G........ 12019

                   ND5
Pigeon (GU908131)  ATTCATCATAAACTTCAAAATCCCAATTAGCCTTAAAATAGATCAATACTCCCTAATATTTCTCCCCGTCGCACTACTTG 12109
JQ692598           G.......................T.................C.................C.....TA.....T..A... 11977
BMNH1149           G.......................T.................C.................C.....TA.....T..A... 12137
BMNH1389           G.......................T.................C.................C.....TA.....T..A... 12099
```

```
                     ND5
Pigeon (GU908131)    TAACGTGATCCATCCTCCAATTCGCAACATGATACATAAATACAGAACCTTATATCACAAAATTCTTCTTCTACCTCCTA 12189
JQ692598             ....A.....T........G...................GC...........C..T.............C.......... 12057
BMNH1149             ....A.....T........G...........G.......GC...........C..T.............C.......... 12217
BMNH1389             ....A.....T........G...................GC...........C..T.............C.......... 12179
                     ND5
Pigeon (GU908131)    CTGTCCCTAATCGCTATACCAACACTAACCATTGCCAACAACATGTTCCTTCTATTCATTGGCTGAGAAGGCGTCGGAAT 12269
JQ692598             A.A.T.........C...TT..........G.C..T...G....A..............C...........A........ 12137
BMNH1149             A.A.T.........C...TT..........G.C..T...G....A..............C...........A........ 12297
BMNH1389             A.A.T.........C...TT..........G.C..T...G....A..............C...........A........ 12259
                     ND5
Pigeon (GU908131)    CATGTCATTCCTACTAATCGGCTGATGACAGGGCCGAGCAGAAGCTAACACAGCCGCCCTCCAAGCTGTACTCTACAACC 12349
JQ692598             ...A..........................A..A..G...........T...........T........G.......... 12217
BMNH1149             ...A..........................A..A..G...........T...........T........G.......... 12377
BMNH1389             ...A..........................A..A..G...........T...........T........G.......... 12339
                     ND5
Pigeon (GU908131)    GAATTGGAGACATCGGTCTAATCCTAAGTATAGCCTGACTTGCTTCCACTACAAACACCTGAGAAATCCAACAAACAACC 12429
JQ692598             ....C.....T.....C..C..T..G..C...........C........C.T.....................GG.C... 12297
BMNH1149             ....C.....T.....C..C..T..G..C...........C........C.T.....................GG.C... 12457
BMNH1389             ....C.....T.....C..C..T..G..C...........C........C.T.....................GG.C... 12419
                     ND5
Pigeon (GU908131)    TCTACCACCCAAACACCTATTCTCCCTCTACTAGGCCTTATCCTAGCCGCTACAGGAAAATCCGCCCAATTCGGCCTCCA 12509
JQ692598             ...C.........TC..A.CC.....C..G....................C...........T..............T.. 12377
BMNH1149             ...C.........TC..A.CC.....C..G....................C...........T..............T.. 12537
BMNH1389             ...C.........TC..A.CC.....C..G....................C...........T..............T.. 12499
                     ND5
Pigeon (GU908131)    CCCTTGACTGCCGGCTGCCATAGAAGGCCCAACTCCAGTCTCTGCCTTACTCCACTCCAGCACAATAGTAGTGGCAGGAA 12589
JQ692598             T..C.....A..A..C..T..............C.....T......C...................G.....A....... 12457
BMNH1149             T..C.....A..A..C..T..............C.....T......C...................G.....A....... 12617
BMNH1389             T..C.....A..A..C..T..............C.....T......C...................G.....A....... 12579
                     ND5
Pigeon (GU908131)    TCTTCCTACTCATCCGTACCCACCCACTACTAGCCACTAACCAAACCGCCCTAACCCTATGTCTATGCCTAGGAGCCCTA 12669
JQ692598             ..........G.....C..A..T.........A...AC........T.....T........C..C...T....G.....C 12537
BMNH1149             ..........G.....C..A..T.........A...AC........T.....T........C..C...T....G.....C 12697
BMNH1389             ..........G.....C..A..T.........A...AC........T.....T........C..C...T....G.....C 12659
                     ND5
Pigeon (GU908131)    TCAACACTATTCGCCGCAACATGTGCCCTCACACAAAATGACATCAAAAAAATCATTGCCTTCTCTACATCAAGCCAACT 12749
JQ692598             ..C........T..T...........A..T........C.....T..........................C........ 12617
BMNH1149             ..C........T..T...........A..T........C.....T..........................C........ 12777
BMNH1389             ..C........T..T...........A..T........C.....T..........................C........ 12739
```

```
                  ND5
Pigeon (GU908131) AGGCCTAATAATAGTTACAATCGGACTAAACCTCCCACAACTAGCCTTCCTGCATATCTCAACCCACGCTTTCTTCAAAG 12829
         JQ692598 .....................T...........T.....G..G.....T..A..C........T.....C.......... 12697
         BMNH1149 .....................T...........T.....G..G.....T..A..C..............C.......... 12857
         BMNH1389 .....................T...........T.....G..G.....T..A..C........T.....C.......... 12819
                  ND5
Pigeon (GU908131) CAATACTCCTCCTATGTTCAGGATCCATCATCCACAGTCTCAATGGAGAACAGGACATCCGGAAAATAGGGTGCCTACAA 12909
         JQ692598 .C..G...T....C..C........T........T..C..T..C........A........A........AGC.T..... 12777
         BMNH1149 .C..G...T....C..C........T........T..C..T..C........A........A........AGC.T..... 12937
         BMNH1389 .C..G...T....C..C........T........T..C..T..C........A........A........AGC.T..... 12899
                  ND5
Pigeon (GU908131) AAAATCCTACCAACAACCACCTCCTGCCTGACTATTGGTAACCTAGCACTAATAGGAACCCCATTCCTAGCAGGATTTTA 12989
         JQ692598 ......T....C...............T.A..C..............C.....G.....T.....T...........C.. 12857
         BMNH1149 ......T....C...............T.A..C..............C.....G.....T.....T...........C.. 13017
         BMNH1389 ......T....C...............T.A..C..............C.....G.....T.....T...........C.. 12979
                  ND5
Pigeon (GU908131) TTCAAAAGATCTCATCATCGAAAACCTAAATACATCTTACCTAAACACCTGAGCACTCCTCCTAACCCTCCTAGCCACAT 13069
         JQ692598 C........CT.A.................C.....A..............G...T.G...................... 12937
         BMNH1149 C........CT.A.................C.....A..............G...T.A...................... 13097
         BMNH1389 C........CT.A.................C.....A..............G...T.G...................... 13059
                  ND5
Pigeon (GU908131) CCTTCACCGCAACCTACACCCTTCGCATAACCTTACTAGTCCAAACAGGATTCACCCGTACACCCTCAATCACCCCAGCA 13149
         JQ692598 ....T........A........C........TC.T.....T...........T..T........T............... 13017
         BMNH1149 ....T........A........C........TC.T.....T...........T..T..C.....T............... 13177
         BMNH1389 ....T........A........C........TC.T.....T...........T..T........T............... 13139
                  ND5
Pigeon (GU908131) AATGAAAACGACCAAGCAGTCACCGCCCCCATCACCCGACTTGCATTGGGCAGCATTATAGCAGGCCTAATCATCACATC 13229
         JQ692598 ..C.....TA..........T........A..............C..A..T........C.....A...C.......... 13097
         BMNH1149 ..C.....TA..........T........A..............C..A..T........C.....A...C.......... 13257
         BMNH1389 ..C.....TA..........T........A..............C..A..T........C.....A...C.......... 13219
                  ND5
Pigeon (GU908131) CTACATCCTCCCCACAAAAACCCCTCCCATAACCATACCTACAACCACAAAAACTGCTGCTATTATCATCACAATCTTAG 13309
         JQ692598 ......T..............A..A..............A..T.T.........C..C..C...C..G.......TC... 13177
         BMNH1149 ......T..............A..A..............A..T.T.........C..C..C...C..G.......TC... 13337
         BMNH1389 ......T..............A..A..............A..T.T.........C..C..C...C..G.......TC... 13299
                  ND5
Pigeon (GU908131) GTGCCATCCTAGCCCTCGAACTCTCCAACATAACACACATACTAACCCACCCCAAACAAAACAGCCTCCTAAATTTCTCC 13389
         JQ692598 .AATT...T.......A.....T.................G.......T...T..........A....T....C.....T 13257
         BMNH1149 .AATT...T.......A.....T.................G.......T...T..........A....T....C.....T 13417
         BMNH1389 .AATT...T.......A.....T.................G.......T...T..........A....T....C.....T 13379
```

	ND5		
Pigeon (GU908131)	TCCTCCCTAGGCTTCTTTAACCCATTAACCCACCGACTTAGCTCAACAGGCCTCCTACTCTCCGGACAAAAAATCGCCTC	13469
JQ692598	......T......A...C.....CC.......T.....C.......T.A....A....................T.....	13337
BMNH1149	......T......A...C.....CC.......T.....C.......T.A....A....................T.....	13497
BMNH1389	......T......A...C.....CC.......T.....C.......T.A....A....................T.....	13459
	ND5		
Pigeon (GU908131)	CCACCTAATTGACTTATCCTGATACAAAAAAATAGGCCCTGAAGGACTCGCAGACCTCCAACTCATAGCAGCCAAAACCT	13549
JQ692598	A........C...C..................................T.....T..T.........ATGA.T.......	13417
BMNH1149	A........C...C..................................T.....T..T.........ATGA.T.......	13577
BMNH1389	A........C...C..................................T.....T..T.........ATGA.T.......	13539
	ND5		
Pigeon (GU908131)	CAATCAACCTTCACACCGGCCTAATCAAAGCCTACCTGGAATCCTTCGCTCTATCCATTCTCATCATTCTATCCCTCCAT	13629
JQ692598	..........C.....T..T.................A...........A........C..............T......	13497
BMNH1149	..........C.....T..T.................A...........A........C..............T......	13657
BMNH1389	..........C.....T..T.................A...........A........C..............T......	13619
	ND5 / CYTB		
Pigeon (GU908131)	AGACCTGTAAACCAATGGCCCCCGATCTACGAAAATACCACCCTCTACTAAAAATAATCAATAACTCCCTAATCGACCTA	13709
JQ692598	.....C.-...............A.CT....G...C.......C............G.T..C...........T......	13576
BMNH1149	.....C.-...............A.CT....G...C.......C............G.T..C...........T......	13736
BMNH1389	.....C.-...............A.CT....G...C.......C............G.T..C...........T......	13698
	CYTB		
Pigeon (GU908131)	CCAACCCCCTCAAACATCTCCGCCTGATGGAACTTTGGGTCCCTACTAGGCATTTGCTTGCTAACTCAAATCCTAACCGG	13789
JQ692598	.....T.....T........T........A........A..T........A..C...C.A.................T..	13656
BMNH1149	.....T.....T........T........A........A..T........A..C...C.A.................T..	13816
BMNH1389	.....T.....T........T........A........A..T........A..C...C.A.................T..	13778
	CYTB		
Pigeon (GU908131)	CTTACTACTCGCCGCACATTACACTGCTGACACCACCCTAGCCTTTTCATCCGTTGCACACACATGCCGAAACGTACAGT	13869
JQ692598	.C................C........A.....A...........C........C...........T...........A.	13736
BMNH1149	.C................C........A.....A...........C........C...........T...........A.	13896
BMNH1389	.C................C........A.....A...........C........C...........T...........A.	13858
	CYTB		
Pigeon (GU908131)	ACGGCCGGCTAATCCGAAACCTCCATGCAAACGGGGCCTCATTTTTCTTCATCTGTATTTACCTACACATCGGACGAGGA	13949
JQ692598	.....T.A........C...T.A..C........A..T.....C...........C..C..T..C...............	13816
BMNH1149	.....T.A........C...T.A..C........A..T.....C...........C..C..T..C...............	13976
BMNH1389	.....T.A........C...T.A..C........A..T.....C...........C..C..T..C...............	13938
	CYTB		
Pigeon (GU908131)	CTCTACTACGGATCCTACCTCTACAAAGAGACTTGAAACACAGGAGTCGTCCTCCTACTAACCCTTATAGCCACTGCATT	14029
JQ692598	.....T..T.....T.....A........A.................T.....T....................A..C..	13896
BMNH1149	.....T..T..G..T.....A........A.................T.....T....................A..C..	14056
BMNH1389	.....T..T.....T.....A........A.................T.....T........T...........A..C..	14018

```
CYTB
Pigeon (GU908131) CGTAGGATATGTCCTACCCTGAGGACAAATATCATTCTGAGGAGCTACAGTCATTACCAATCTATTCTCAGCTGTCCCCT 14109
JQ692598 T............................................C...........T..C........G.......... 13976
BMNH1149 T............................................C...........T..C........G.......... 14136
BMNH1389 T............................................C...........T..C........G.......... 14098
CYTB
Pigeon (GU908131) ACATTGGCCAAACCCTCGTTGAATGAGCCTGAGGCGGATTTTCCGTAGATAACCCTACATTAACACGATTCTTCACCCTT 14189
JQ692598 ....C..T.....T..T..C.....G........T..C..C..A..C..C.................G............ 14056
BMNH1149 ....C..T.....T..T..C.....G........T..C..C..A..C..C.................G............ 14216
BMNH1389 ....C..T.....T..T..C.....G........T..C..C..A..C..C.................G............ 14178
CYTB
Pigeon (GU908131) CACTTCCTCCTCCCCTTTATAATCGCAGGCCTCACCATCATCCACCTCACCTTCCTGCACGAATCAGGCTCAAACAACCC 14269
JQ692598 ........T........C.....T...........T..T..T..T...........A..T.....T.............. 14136
BMNH1149 ........T........C.....T...........T..T..T..T...........A..T.....T.............. 14296
BMNH1389 ........T........C.....T...........T..T..T..T...........A..T.....T.............. 14258
CYTB
Pigeon (GU908131) ACTAGGCATCACCTCCAACTGCGATAAAATCCCATTCCACCCCTACTTCTCCCTAAAAGACATCCTCGGCTTCATGGTAA 14349
JQ692598 .........TT..........T..C...........................T.............T........AC... 14216
BMNH1149 .........TT..........T..C...........................T.............T........AC... 14376
BMNH1389 .........TT..........T..C...........................T.............T........AC... 14338
CYTB
Pigeon (GU908131) TACTCCTCCCCCTAATGACCCTAGCCCTATTCTCCCCCAACCTTCTAGGAGACCCAGAAAACTTCACGCCTGCAAACCCT 14429
JQ692598 ..T.............A..A..............T........CT....G...........T.....A..C........A 14296
BMNH1149 ..T.............A..A..............T........CT....G...........T.....A..C........A 14456
BMNH1389 ..T.............A..A..............T........CT....G...........T.....A..C........A 14418
CYTB
Pigeon (GU908131) CTAGTTACACCTCCCCATATCAAACCAGAGTGATACTTCCTATTCGCATACGCCATCCTACGCTCCATCCCCAATAAACT 14509
JQ692598 .....A........A..C..T..G..T..A..G..T........T.......................T.....C..... 14376
BMNH1149 .....A........A..C..T..G..T..A..G..T........T.............................C..... 14536
BMNH1389 .....A........A..C..T..G..T..A..G..T........T.............................C..... 14498
CYTB
Pigeon (GU908131) AGGCGGAGTACTAGCCTTAGCCGCCTCAGTACTAATTCTATTCCTCACCCCCCTACTCCACAAGTCCAAACAACGCACAA 14589
JQ692598 ...............AC....T.....C........C...............T.C..T..T..A..T........T.... 14456
BMNH1149 ...............AC....T.....C........C...............T.C..T..T..A..T........T.... 14616
BMNH1389 ...............AC....T.....C........C...............T.C..T..T..A..T........T.... 14578
CYTB
Pigeon (GU908131) TAATCTTCCGCCCACTCTCTCAACTCCTATTCTGAATCCTAGTCACCAACCTCCTTATCCTAACATGAGTTGGAAGCCAA 14669
JQ692598 .......T.....C......................................T..C..............A..G...... 14536
BMNH1149 .......T.....C......................................T..C..............A..G...... 14696
BMNH1389 .......T.....C......................................T..C..............A..G...... 14658
```

```
                      CYTB
Pigeon (GU908131)     CCTGTAGAACACCCCTTCATCATTATCGGCCAACTAGCCTCCCTCACCTACTTCACCATCCTCCTTGTCCTCTTCCCTGC 14749
JQ692598              . . . . . . . . . . . . . . A . . . . . . . . C . . . . . T . . . . . . . . . . . T . . . . . . . . . . . . . T . . . . . . . . . . A . . . . . . . . . . C . . 14616
BMNH1149              . . . . . . . . . . . . . . A . . . . . . . . C . . . . . T . . . . . . . . . . . T . . . . . . . . . . . . . T . . . . . . . . . . A . . . . . . . . . . C . . 14776
BMNH1389              . . . . . . . . . . . . . . A . . . . . . . . C . . . . . T . . . . . . . . . . . T . . . . . . . . . . . . . T . . . . . . . . . . A . . . . . . . . . . C . . 14738
                      CYTB                                  tRNA-Thr
Pigeon (GU908131)     TACCGCAGCCCTAGAAAACAAACTACTTAACTACTAAACTCTAATAGTTTATGAAAACATCGGTCTTGTAAACCGAAGAA 14829
JQ692598              . . T . . G . . . . . . . . . . . . . . . . . . . . C . . . . . . . . . . . . . . . . . . . . . . . . A . . . . . . . . . . . . . . . . . . . . . . . . . . . 14696
BMNH1149              . . T . . G . . . . . . . . . . . . . . . . . . . . C . . . . . . . . . . . . . . . . . . . . . . . . A . . . . . . . . . . . . . . . . . . . . . . . . . . . 14856
BMNH1389              . . T . . G . . . . . . . . . . . . . . . . . . . . C . . . . . . . . . . . . . . . . . . . . . . . . A . . . . . . . . . . . . . . . . . . . . . . . . . . . 14818
                      tRNA-Thr                                                                     tRNA-Pro
Pigeon (GU908131)     TGAAGGCCACTCCCCTTCTTAGAGTTTGCCGC- - -TCGGAAAAGAGGGAATTAAACCCTCACCTCCAACTCCCAAAGCTG 14906
JQ692598              . . . . . . . T . - . . . . . . . . . . . . . . . . . C . . . . CAA . . A . . . . . A . . . . G . . . . . . . . . . . . . . . . . G . . . . . . . . . . . . 14775
BMNH1149              . . . . . . . T . - . . . . . . . . . . . . . . . . . C . . . . CAA . . A . . . . . A . . . . G . . . . . . . . . . . . . . . . . G . . . . . . . . . . . . 14935
BMNH1389              . . . . . . . T . - . . . . . . . . . . . . . . . . . C . . . . CAA . . A . . . . . A . . . . G . . . . . . . . . . . . . . . . . G . . . . . . . . . . . . 14897
                                                    tRNA-Pro                                          ND6
Pigeon (GU908131)     GTATTCTACATTAAACTATCTTCTGATATC- ACACCTCCCTACACCGCCCGAATCGCACTATCACACCTCCCTACACCGC 14985
JQ692598              A . . . . . . T . . . . . . . . . . . T . . . . . . C . C . C . . . . . . . . . . . . . . . . . . . . . . T . . . . MCC . . . . . . . . . . . . . . . . . . 14855
BMNH1149              A . . . . . . T . . . . . . . . . . . T . . . . . . C . C . C . . . . . . . - - - - - - - - - - - - - - - - - - - - - - - - - - - - - - - - - - - - - - - - - - - . . . . . . . . . 14983
BMNH1389              A . . . . . . T . . . . . . . . . . . T . . . . . . C . C . C . . . . . . . - - - - - - - - - - - - - - - - - - - - - - - - - - - - - - - - - - - - - - - - - - - . . . . . . . . . 14945
                                                                                                      ND6
Pigeon (GU908131)     CCGAATCGCACCACGAGACAATCCACGAACAACCTCTAACACCACAAATAACGTAAGCAACAACCCTCACCCCGCCACCA 15065
JQ692598              . . . . . . T . . . . . C . . . . . . . . C . . C . . . . . . . . . . . . . . . . A . . . . . C . . . . . . . . . . . . . . . . . C . . . . . T . . . . . . . 14935
BMNH1149              . . . . . . T . . . . . C . . . . . . . . C . . C . . . . . . . . . . . . . . . . A . . . . . C . . . . . . . . . . . . . . . . . C . . . . . T . . . . . . . 15063
BMNH1389              . . . . . . T . . . . . C . . . . . . . . C . . C . . . . . . . . . . . . . . . . A . . . . . C . . . . . . . . . . . . . . . . . C . . . . . T . . . . . . . 15025
                                                                                                      ND6
Pigeon (GU908131)     GAAACATCCCGACCCCACAAGAATAAAACATAGCTACACCACTAAAATCCAACCGGACAGAAAACATCCCTCCACCATCA 15145
JQ692598              A . . . . . . . . . AG . . . . . TGT . . . . . . . . . . . . . . . . . C . . . . . . . . . . . . . . . . . A . . . . . . . G . . . . . . . . . . . T . . . . 15015
BMNH1149              A . . . . . . . . . AG . . . . . TGT . . . . . . . . . . . . . . . . . C . . . . . . . . . . . . . . . . . A . . . . . . . G . . . . . . . . . . . T . . . . 15143
BMNH1389              A . . . . . . . . . AG . . . . . TGT . . . . . . . . . . . . . . . . . C . . . . . . . . . . . . . . . . . A . . . . . . . G . . . . . . . . . . . T . . . . 15105
                                                                                                      ND6
Pigeon (GU908131)     ACAGTCTCTACCCCCAACTTCCACCCCTCAACAAAACCCCCAACAACAACCCCTACAACCAATACCACAACAAACCCCAA 15225
JQ692598              . . . . . TA . C . . . . . AG . T . . T . . . . . T . . . . . . . . . A . AA . . . . . . . . . . AA . A . . . . . A . CA . . A . . . . . . . . . . . . . . 15095
BMNH1149              . . . . . TA . C . . . . . AG . T . . T . . . . . T . . . . T . . . C . . T . . . G . . . . C . . T . . C . . . . . T . . C . . . . T . . . . . . . . . . . G 15223
BMNH1389              . . . . . TA . C . . . . . AG . T . . T . . . . . T . . . . T . . . C . . T . . . G . . . . C . . T . . C . . . . . T . . C . . . . T . . . . . . . . . . . G 15185
                                                                                                      ND6
Pigeon (GU908131)     ACTATAGCCTACAACACGCCAATCATCTCAAGCCTCAGGAAAAGGATCCGCTGCCAAAGACACCGAGTACACAAACACCA 15305
JQ692598              . . C . . . C . . C . T . . . . . . . . . . . . . CC . C . . . . TT . . . . . G . . C . . G . . T . . . . . . . . . . . . . . . . . A . . . . . . . . . . . . 15175
BMNH1149              . . C . . . C . . C . T . . . . . . . . . . . . . CC . C . . . . TT . . . . . G . . C . . G . . T . . . . . . . . . . . . . . . . . A . . . . . . . . . . . . 15303
BMNH1389              . . C . . . C . . C . T . . . . . . . . . . . . . CC . C . . . . TT . . . . . G . . T . . G . . T . . . . . . . . . . . . . . . . . A . . . . . . . . . . . . 15265
```

```
                                                                                                                 ND6
Pigeon (GU908131) CCAACATGCCCCCCAAATATACCATAAATAGAACAAGTGACATAAATGACACACCCAAACTTAGCAACCAACCGCAACCA 15385
         JQ692598 .T.....A.....T.....C..T..............A....C...C..T.....T.G.....A..G...C..A..C... 15255
         BMNH1149 .T.....A.....T.....C..T..............A....C...C..T.....T.G.....A..G...C..A..C... 15383
         BMNH1389 .T.....A.....T.....C..T..............A....C...C..T.....T.G.....A..G...C..A..C... 15345
                                                                                                 ND6
Pigeon (GU908131) GTGACAGATGCCACAACCAACCCAACAACCCCATAATAAGGAGAAGGATTAGACGCCATCCCTAACCCCCCCAAAACAAA 15465
         JQ692598 .AA.T.........T.......................C...................C...C.....G........... 15335
         BMNH1149 .AA.T.........T.......................C...................C...C.....G........... 15463
         BMNH1389 .AA.T.........T.......................C...................C...C.....G........... 15425
                                                ND6                                       tRNA-Glu
Pigeon (GU908131) ACTAAACCCCAAAAAAAACACAAAGTAAGTCATAGCAGTTTCCGCTTGGCCTTTCTCCAAGGACTGCGGCCTGAAAAGCC 15545
         JQ692598 ..C....AAA..........A...AA...........A....T.......T..........A...A..A.T......A.. 15415
         BMNH1149 ..C......T.......GA.....A............A....T.......T..........A...A..A.T......A.. 15543
         BMNH1389 ..C......T.......GA.....A............A....T.......T..........A...A..A.T......A.. 15505
                               tRNA-Glu|Control region
Pigeon (GU908131) GCCGTTGTAAACCTCAACTACAGAAACTAACGAAAAACACAGGACACAATGCAGGACCC-----CCCCTACCCCCCT--- 15617
         JQ692598 .T..........T........................T.GGAC...-............TCCCC............C--- 15491
         BMNH1149 .T..........T........................T.GGAC...-............TCCCC............CTAC 15622
         BMNH1389 .T..........T........................T.GGAC...-............TCCCC............CTAC 15584
                  Control region
Pigeon (GU908131) ------GCATTTGTGTCCTATGTACTACAGTGCATCGATTTATTTACCATATTCATGACCCCCATACGTATTAAAGTCAA 15691
         JQ692598 ------....................G................................................AC..- 15564
         BMNH1149 CCCCCC....................G................................................AC..- 15701
         BMNH1389 CCCCCC....................G................................................AC..- 15663
                  Control region
Pigeon (GU908131) CATGTAATGTAGTCACATGATCCACATCTATGTACCCAGGCATATTATCTATT-TCCAGGTTCCATATCCACATAATCCT 15770
         JQ692598 ...C........AG..TAA..A..T.AT......A..........C....C.CGC..C.AC.ATCCCC..C...G.AATG 15644
         BMNH1149 ...C........AG..TAA..A..T.AT......A..........C....C.CGC..C.AC.ATCCCC..C...G.AATG 15781
         BMNH1389 ...C........AG..TAA..A..T.AT......A..........C....C.CGC..C.AC.ATCCCC..C...G.AATG 15743
                  Control region
Pigeon (GU908131) CAAACGATCCATTAATATGTCCATGTATTAAGGCATATATGTAGACGAGC-TATAACCTCCCCGACGCATTC-TCCGTCC 15848
         JQ692598 ......T...TC...-..A.G...TC.CCT..A....A.CCCCT..C..AA..GC.G..TTTTAC.A...CTA...T... 15723
         BMNH1149 ......T...TC...-..A.G...TC.CCT..A....A.CCCCT..C..AA..GC.G..TTTTAC.A...CTA...T... 15860
         BMNH1389 ......T...TC...-..A.G...TC.CCT..A....A.CCCCT..C..AA..GC.G..TTTTAC.A...CTA...T... 15822
                  Control region
Pigeon (GU908131) AGAGGACTAGAACTTAATG-ATACCTTAGACATAATAGGTTATTACTTCGTACTAAACCCATGGAAAGCCAGTTTGTGCA 15927
         JQ692598 ....T..CCA..A.G..CCC.CGTA.C..G....TCCCA.A..A..AC..............ACCT..TA.....A.... 15803
         BMNH1149 ....T..CCA..A.G..CCC.CGTA.C..G....TCCTA.A..A..AC..............ATCT..TA.....A.... 15940
         BMNH1389 ....T..CCA..A.G..CCC.CGTA.C..G....TCCCA.A..A..AC..............ACCT..TA.....A.... 15902
```

Control region

Pigeon (GU908131)	TACCCTCAATATCCATACGGAAGTGCCCTAGTACAGACTATGCTTGGTGTAGTCCATAACATGAGATATCTCCTGAAGTA	16007
JQ692598	..A.TC...C-..A.C....C.....T.GCA....C....C.......ACC.C....CC.-.....A.............	15881
BMNH1149	..A.TC...C-..A.C....C.....T.GCA....T....C.......ACC.C....CC.-.....A.............	16018
BMNH1389	..A.TC...C-..A.C....C.....T.GCA....C....C.......ACC.C....CC.-.....A.............	15980

Control region

Pigeon (GU908131)	CATAAAGCAGGGACCAGGTTATCTATTAATCTTACACCTCACGTGAAACCAGCAACTCGACGCGAGAAGTATCCATCACG	16087
JQ692598	......................T...	15961
BMNH1149	......................T...	16098
BMNH1389	......................T...	16060

Control region

Pigeon (GU908131)	ACTAGCTTCAGGCCCATTCTTCCCCCCTACACCCTAGCACGACTTGCTCTTTTGCGCCTCTGGTTCCTATGTCAGGGCCA	16167
JQ692598	.....................T..CG..........	16041
BMNH1149	.....................T..CG..........	16178
BMNH1389	.....................T..CG..........	16140

Control region

Pigeon (GU908131)	TAACTTGCCAATTCCCATGACCTCGCTCTTCACAGATACATCTGGTCGGGGTCATACCTCACCATTTCAGTCCGTGATCG	16247
JQ692598	.T..C..TT..C...T....T..T..C...	16121
BMNH1149	.T..C..TT..C...T....T..T..C...	16258
BMNH1389	.T..C..TT..C...T....T..T..C...	16220

Control region

Pigeon (GU908131)	CGGCATTTCCCCGACCTTGGCGCCTTTGGTTTT-TTTTCTCTCTCTCTCCCGCAGCTCGCCCCTCAAGTGCGGCGGGCAC	16326
JQ692598	.......-.......................C.C................T....-...............AA....TGA	16199
BMNH1149	.......-.......................C.C................T....-...............AA....TGA	16336
BMNH1389	.......-.......................C.C................T....-...............AA....TGA	16298

Control region

Pigeon (GU908131)	ATTGGTTTATATTCTGCACCTAAATTATGCGTTACCAACTAATCTCGACCTCAGGTACTACTGGCGTTACGGCTTAA-AG	16405
JQ692598	............C.....................T..C..........T.......C..G............T....CG.	16279
BMNH1149	............C.....................T..C..........T.......C..G............T....CG.	16416
BMNH1389	............C.....................T..C..........T.......C..G............T....CG.	16378

Control region

Pigeon (GU908131)	ATAACCGGTATCACCTTGACACTGATGCACTTTGTCTTCCATAACTCGGCTGGATGTAATGGATTAAGGACATACAGAGC	16485
JQ692598	....G....TA.........TC..........T.....	16359
BMNH1149	....G....TA.........TC..........T.....	16496
BMNH1389	....G....TA.........TC..........T.....	16458

Control region

Pigeon (GU908131)	TTCGCCCGCGAGATGCACCCTTTCGAGCATCTGGTTATGGTGTGTCCGCAAGTACCTACAAATGCTGCATATTAGTGAAT	16565
JQ692598	C.YTTTTTTTT.T.TTTTTT...TTTTTT.T.....T...--	16400
BMNH1149	C..C......C........TCCCTA.A....................A....CT..........T.A..CTCC...T...	16576
BMNH1389	C..C......C........TCCCTA.A....................A....CT..........T.A..CTCC...T...	16538

```
                     Control region
Pigeon (GU908131)    GCTCGCAGGACATAAATTTCCACCATTTTACCCTATTTACTTCCTCTAACTTTCTAAGCAACACGGCTAACTTTCAACTA  16645
JQ692598             --------------------------...TTTT.T...TT..TT.T.TTT...T--------------------------  16428
BMNH1149             ...T...............T.....A...C..A...C..T.C...........TA..A.........C.......T.AC.  16656
BMNH1389             ...T...............T.....A...C..A...C..T.C...........TA..A.........C.......T.AC.  16618
                     Control region
Pigeon (GU908131)    AACACTCAAAATACCGACCCAAAATCTTGTAAATTTCACTTTCTTTTTCTTTTTTTTTCCTATGATTACCACTGGAGTTC  16725
JQ692598             --------------------------..T.TTT...TTT...T.....TC........TT.T.TT..TTTTT.T.TT..T  16482
BMNH1149             ...G.C..-----------T.G.CG..C..TTG..GTC.G..TG..G.T.G...G..GTT.TGTT.GTTTGT.T.TT.GT  16725
BMNH1389             ...G.C..-----------T.G.CG..C..TTG..GTC.G..TG..NNNNNNNNNNNNNNNNNNNN--------------  16673
                     Control region
Pigeon (GU908131)    CATTAATAATTCATCATACGATTCATACGTACGTATGTTAATCCTCTGACAAACCATTAATAACTCATCAAATTTTTCCA  16805
JQ692598             TT..TT.TT..TT.TT.TTTT..T------------------------------------------------.....TTT  16514
BMNH1149             ....TG.T...TG.TTGTT.T..G------------------------------------------------...G.TGT  16757
BMNH1389             -------------------------------------------------------------------------------T  16674
                     Control region
Pigeon (GU908131)    TTATTTGTTGTTGATTTTTCATCATTCACCCATCTAATATTAACCGAATTTAGCCACACTTTTCCCATTTTCACTCATCA  16885
JQ692598             .CC...T..T..TT...----------TTTTT.T...C----------------------.WYT.AGC.CAA.AC-----  16557
BMNH1149             ..G.........TGC..GC.TAT.AC.TA.T....TC-----------------------.CC..TG...AT...-----  16810
BMNH1389             ..G.........TGC..GC.TAT.AC.TA.T....TC-----------------------.CC..TG...AT...-----  16727
                     Control region
Pigeon (GU908131)    ATTGTCCAAAACATTAGACCAATTAAGCCACTCTCCTCATCACCCGCTCACTCACCAACTCTTGTCCAAAACATTAGACC  16965
JQ692598             ---------..A.CA.A..A.--------------------------------------------A.....---------  16577
BMNH1149             ---------..A....C.TT...A.-----------------------..TAC..A..TGCTC...AT.C.C--------  16851
BMNH1389             ---------..A....C.TT...A.-----------------------..TAC..A..TGCTC...AT.C.C--------  16768
                     Control region
Pigeon (GU908131)    AATTTAAGCCACTCTCCTCATCACCCGCTCACTCACCAACTCTTGTCCAAAACATTA--GACCAATTTAAGCCACTCTCC  17043
JQ692598             -------------------------------AAA..A..ACAAAAAA.......AA.ACA.A...ACA..AA--------  16618
BMNH1149             -------------------------------.A..TT..TC..CTAA....C.....AC..G.T......TT.T...C.T  16900
BMNH1389             -------------------------------.A..TT..TC..CTAA....C.....AC..G.T......TT.T...C.T  16817
                     Control region
Pigeon (GU908131)    TCATCACCCGCTCACTCACCAACTCTTGTCCAAAACATTAGACCAATTTAAGCCGTACAAGTAACCGCCGAAAAACAAAC  17123
JQ692598             --------------------...-------------------------------------------------........  16629
BMNH1149             C.CCTTA..A.A.C.C.T...C.-------------------------------------------------..C...CT  16931
BMNH1389             C.CCTTA..A.A.C.C.T...C.-------------------------------------------------..C...CT  16848
                     Control region
Pigeon (GU908131)    AAACAAACAAACAAACAAACAAACAAACAAACAAACAAACAAACAAACAAACAAACAAACAAACAAACAAACAAACAAAC  17203
JQ692598             ...................A..----.........A..--.C.A.C.--...............................  16701
BMNH1149             T...CG.GC...C.....CA..C--.........CA..CA.C.A.C.GC..AC.G.......TA..CA.T.G.T.GT..A  17009
BMNH1389             T...CG.GC...C.....CA..C--..NNNNNNNNNNNNNNNNNNNNNN..AC.G.......TA..CAGT.G.T.GT..A  16926
```

Control region

```
Pigeon (GU908131)  AAACAAACAAACAAACAAACAAACAC  17229

JQ692598           ...........---.---------..  16715

BMNH1149           C.G...GT....C..---------..  17026

BMNH1389           C.G...GT....C..---------..  16943
```
