# Supplementary material for: The De Novo Assembly of Mitochondrial Genomes of the Extinct Passenger Pigeon (Ectopistes migratorius) with Next Generation Sequencing
Source: PLoS One. 2013 Feb 20;8(2):e56301. doi: 10.1371/journal.pone.0056301 (PMC3577829; doi:10.1371/journal.pone.0056301)
Supplement: Table S1 — Mitogenomic divergence between the rock pigeon ( Columba livia , GenBank accession number GU908131) and each of the three passenger pigeons (one unpublished sequence with a GenBank accession number JQ692598 and two sequenced in this study). We divided the mitogenome into regions encoding 22 transfer RNA genes (Phenylalanine [tRNA-Phe], Valine [tRNA-Val], Leucine [tRNA-Leu], Isoleucine [tRNA-Ile], Glutamine [tRNA-Gln], Methionine [tRNA-Met], Tryptophan [tRNA-Trp], Alanine [tRNA-Ala], Asparagine [tRNA-Asn], Cysteine [tRNA-Cys], Tyrosine [tRNA-Tyr], Serine [tRNA-Ser], Asparate [tRNA-Asp], Lysine [tRNA-Lys], Glycine [tRNA-Gly], Arginine [tRNA-Arg], Histidine [tRNA-His], Serine [tRNA-Ser], Leucine [tRNA-Leu], Threonine [tRNA-Thr], Proline [tRNA-Pro], Glutamate [tRNA-Glu]), 13 protein-coding genes (NADH dehydrogenase subunit 1 [ND1], NADH dehydrogenase subunit 2 [ND2], cytochrome c oxidase subunit I [COX1], cytochrome c oxidase subunit II [COX2], ATP synthase F0 subunit 8 [ATP8], ATP synthase F0 subunit 6 [ATP6], cytochrome c oxidase subunit III [COX3], NADH dehydrogenase subunit 3 [ND3], NADH dehydrogenase subunit 4L [ND4L], NADH dehydrogenase subunit 4 [ND4], NADH dehydrogenase subunit 5 [ND5], cytochrome b [CYTB], NADH dehydrogenase subunit 6 [ND6]), two ribosomal RNA genes (12 s rRNA and16 s rRNA), a control region and the remaining intergenic noncoding regions. For each of these regions, its alignment length (bp) and number of segregating sites/number of gaps/number of ambiguous sites are shown. Numbers that are extraordinarily high or low were bolded. (DOCX) [file pone.0056301.s002.docx]

|  | tRNA-Phe | 12S rRNA | | tRNA-Val | | 16S rRNA | | tRNA-Leu | | ND1 | | tRNA-Ile | | tRNA-Gln | |  |  |
| --- | --- | --- | --- | --- | --- | --- | --- | --- | --- | --- | --- | --- | --- | --- | --- | --- | --- |
| Alignment length | 70 | 975 | | 73 | | 1633 | | 74 | | 966 | | 71 | | 71 | |  |  |
| GU908131/BMNH1149 | 7/2/0 | 80/5/0 | | 5/1/0 | | 155/**36**/20 | | 3/0/0 | | 132/0/0 | | 2/0/0 | | 4/0/0 | |  |  |
| GU908131/BMNH1389 | 7/2/0 | 80/5/0 | | 5/1/0 | | 153/**58**/0 | | 3/0/0 | | 131/0/0 | | 2/0/0 | | 4/0/0 | |  |  |
| GU908131/JQ692598 | 8/3/0 | 80/5/0 | | 5/1/0 | | 162**/176/**0 | | 3/0/0 | | 131/0/0 | | 2/0/0 | | 4/0/0 | |  |  |
|  |  |  | |  | |  | |  | |  | |  | |  | | |  |
|  |  |  | |  | |  | |  | |  | |  | |  | | |  |
|  | tRNA-Met | ND2 | | tRNA-Trp | | tRNA-Ala | | tRNA-Asn | | tRNA-Cys | | tRNA-Tyr | | COX1 | |  |  |
| Alignment length | 69 | 1042 | | 71 | | 69 | | 72 | | 67 | | 72 | | 1551 | |  |  |
| GU908131/BMNH1149 | 2/0/0 | 162/1/0 | | 3/0/0 | | 6/0/0 | | 4/0/0 | | 1/0/0 | | 2/0/0 | | 162/0/0 | |  |  |
| GU908131/BMNH1389 | 2/0/0 | 161/1/0 | | 3/0/0 | | 6/0/0 | | 4/0/0 | | 1/0/0 | | 2/0/0 | | 163/0/0 | |  |  |
| GU908131/JQ692598 | 2/0/0 | 162/1/0 | | 3/0/0 | | 6/0/0 | | 4/0/0 | | 1/0/0 | | 2/0/0 | | 162/0/0 | |  |  |
|  |  | |  | |  | |  | |  | |  | |  | |  | | |
|  |  | |  | |  | |  | |  | |  | |  | |  | | |
|  | tRNA-Ser | | tRNA-Asp | | COX2 | | tRNA-Lys | | ATP8 | | ATP6 | | COX3 | | tRNA-Gly |  |  |
| Alignment length | 74 | | 69 | | 684 | | 71 | | 168 | | 684 | | 784 | | 69 |  |  |
| GU908131/BMNH1149 | 1/0/0 | | 0/0/0 | | 87/0/0 | | 8/0/0 | | 24/0/0 | | 96/0/0 | | 79/0/0 | | 2/0/0 |  |  |
| GU908131/BMNH1389 | 1/0/0 | | 0/0/0 | | 87/0/0 | | 8/0/0 | | 24/0/0 | | 96/0/0 | | 80/0/0 | | 2/0/0 |  |  |
| GU908131/JQ692598 | 1/0/0 | | 0/0/0 | | 87/0/0 | | 8/0/0 | | 24/0/0 | | 96/0/0 | | 79/0/0 | | 2/0/0 |  |  |

|  | ND3 | tRNA-Arg | ND4L | | ND4 | | tRNA-His | | tRNA-Ser | tRNA-Leu | | | ND5 | | |  |  |
| --- | --- | --- | --- | --- | --- | --- | --- | --- | --- | --- | --- | --- | --- | --- | --- | --- | --- |
| Alignment length | 352 | 69 | 297 | | 1378 | | 69 | | 66 | 72 | | | 1815 | | |  |  |
| GU908131/BMNH1149 | 51/0/0 | 2/0/0 | 37/0/0 | | 181/0/0 | | 1/0/0 | | 0/0/0 | 0/1/0 | | | 254/0/0 | | |  |  |
| GU908131/BMNH1389 | 51/0/0 | 2/0/0 | 37/0/0 | | 181/0/0 | | 1/0/0 | | 0/0/0 | 0/1/0 | | | 252/0/0 | | |  |  |
| GU908131/JQ692598 | 51/0/0 | 2/0/0 | 37/0/0 | | 181/0/0 | | 1/0/0 | | 0/0/0 | 0/1/0 | | | 252/0/0 | | |  |  |
|  |  |  |  | |  | |  | |  |  | | |  | | | |  |
|  |  |  |  | |  | |  | |  |  | | |  | | | |  |
|  | CYTB | tRNA-Thr | tRNA-Pro | | ND6 | | tRNA-Glu | | Control region | | Intergenic regions | | |  | | |  |
| Alignment length | 1143 | 69 | 70 | | 522 | | 71 | | 1679 | | 118 | | |  | | |  |
| GU908131/BMNH1149 | 138/0/0 | 2/1/0 | 7/0/0 | | 80/0/0 | | 10/0/0 | | 352/**245**/0 | | 16/46/0 | | |  | | |  |
| GU908131/BMNH1389 | 138/0/0 | 2/1/0 | 7/0/0 | | 80/0/0 | | 10/0/0 | | 307/**329**/0 | | 16/46/0 | | |  | | |  |
| GU908131/JQ692598 | 138/0/0 | 2/1/0 | | 7/0/0 | | 81/0/0 | 10/0/0 | 289**/422**/3 | | | | 19/14/1 | | |  | | |
